# *C9orf72* expansion within astrocytes reduces metabolic flexibility in amyotrophic lateral sclerosis

**DOI:** 10.1093/brain/awz302

**Published:** 2019-10-24

**Authors:** Scott P Allen, Benjamin Hall, Ryan Woof, Laura Francis, Noemi Gatto, Allan C Shaw, Monika Myszczynska, Jordan Hemingway, Ian Coldicott, Amelia Willcock, Lucy Job, Rachel M Hughes, Camilla Boschian, Nadhim Bayatti, Paul R Heath, Oliver Bandmann, Heather Mortiboys, Laura Ferraiuolo, Pamela J Shaw

**Affiliations:** 1 Sheffield Institute for Translational Neuroscience (SITraN), University of Sheffield, 385 Glossop Road, Sheffield S10 2HQ, UK; 2 The Living Systems Institute, University of Exeter, Stocker Road, Exeter, EX4 4QD, UK

**Keywords:** ALS, metabolism, *C9orf72*, astrocytes, methylglyoxal

## Abstract

It is important to understand how the disease process affects the metabolic pathways in amyotrophic lateral sclerosis and whether these pathways can be manipulated to ameliorate disease progression. To analyse the basis of the metabolic defect in amyotrophic lateral sclerosis we used a phenotypic metabolic profiling approach. Using fibroblasts and reprogrammed induced astrocytes from *C9orf72* and sporadic amyotrophic lateral sclerosis cases we measured the production rate of reduced nicotinamide adenine dinucleotides (NADH) from 91 potential energy substrates simultaneously. Our screening approach identified that *C9orf72* and sporadic amyotrophic lateral sclerosis induced astrocytes have distinct metabolic profiles compared to controls and displayed a loss of metabolic flexibility that was not observed in fibroblast models. This loss of metabolic flexibility, involving defects in adenosine, fructose and glycogen metabolism, as well as disruptions in the membrane transport of mitochondrial specific energy substrates, contributed to increased starvation induced toxicity in *C9orf72* induced astrocytes. A reduction in glycogen metabolism was attributed to loss of glycogen phosphorylase and phosphoglucomutase at the protein level in both C9orf72 induced astrocytes and induced neurons. In addition, we found alterations in the levels of fructose metabolism enzymes and a reduction in the methylglyoxal removal enzyme GLO1 in both C9orf72 and sporadic models of disease. Our data show that metabolic flexibility is important in the CNS in times of bioenergetic stress.

## Teaser

Energy metabolism is altered in amyotrophic lateral sclerosis and its animal models. Using metabolic profiling, Allen *et al.* reveal a loss of metabolic flexibility in induced astrocytes derived from patients with *C9orf72* ALS, caused by defects in glycogen, fructose and mitochondrial energy substrate transport.

## Introduction

Amyotrophic lateral sclerosis (ALS) is an incurable adult onset disease with a lifetime risk of 1 in 400. The disease, which involves degeneration of upper and lower motor neurons, causes muscle paralysis and typically death within 2–3 years of symptom onset. Historically, the anti-glutamatergic agent riluzole has been the only available treatment for ALS, increasing survival by on average 3 months (Bensimon *et al.*, 1994). However, the intravenous free radical scavenger edaravone has now also been licensed for use in ALS patients in Japan and the USA (Writing and Edaravone, 2017). It remains crucial to discover more effective approaches to slow down or stop disease progression in ALS and extend, not only life expectancy, but also the quality of life of patients.

One in 10 of all cases of ALS (and of the pathologically-linked syndrome of frontotemporal dementia) are associated with a hexanucleotide repeat expansion (HRE) in *C9orf72*, which contributes to neuronal death via haploinsufficiency, dipeptide repeat protein production and expanded RNA species forming RNA foci (De Jesus-Hernandez *et al.*, 2011; Mori *et al.*, 2013*a*, *b*; Cooper-Knock *et al.*, 2014) and leads to sequestration of the nuclear export adaptor SFSR1 and impaired DNA repair mechanisms (Hautbergue *et al.*, 2017; Walker *et al.*, 2017). *C9orf72* HRE expansions account for a significant proportion of both familial and apparently sporadic ALS (SALS) patients and not unexpectedly, there are number of common pathways of dysfunction shared by both groups of patients including oxidative stress, mitochondrial dysfunction, and motor neuron toxicity *in vitro* when co-cultured with patient derived astrocytes (Meyer *et al.*, 2014; Lopez-Gonzalez *et al.*, 2016; Onesto *et al.*, 2016; Konrad *et al.*, 2017). Furthermore, our group in a recent publication using a metabolic screening approach, identified dysregulation of adenosine metabolism linked to a loss of adenosine deaminase in *C9orf72* and SALS patient-derived induced astrocytes (Allen *et al.*, 2019).

Both patient subgroups display metabolic dysfunction which can play a key role in disease progression as metabolic pathways are clearly vulnerable to the disease process (Haeusler *et al.*, 2016; Tefera and Borges, 2016; De Vos and Hafezparast, 2017; Vandoorne *et al.*, 2018). Energy metabolism alterations including insulin resistance, altered levels of free fatty acids and perturbations in lipid homeostasis including cholesterol metabolism, have been reported both in patients and animal models of ALS (Dupuis *et al.*, 2004, 2008; Fergani *et al.*, 2007; Pradat *et al.*, 2010; Palamiuc *et al.*, 2015). Furthermore, patient hypermetabolism and mitochondrial electron transport chain dysfunction, coupled with increased reactive oxygen species, can lead to mitochondrial uncoupling and reduced ATP output. These metabolic alterations are observed not only in the CNS, but also in peripheral tissues (Allen *et al.*, 2003, 2014; Ferraiuolo *et al.*, 2011*b*; Bartolome *et al.*, 2013; Raman *et al.*, 2015; Tefera and Borges, 2016). Fibroblasts are now established as a robust initial model for studying ALS patient subgroups. Mitochondrial dysfunction, including altered respiratory chain activity and increased uncoupling, glycolytic upregulation and metabolic gene expression changes, have been recorded in fibroblasts grown in culture from sporadic and familial ALS cases (Allen *et al.*, 2014, 2015; Kirk *et al.*, 2014; Raman *et al.*, 2015). Fibroblasts isolated from ALS patients with a SOD1 mutation have reduced mitochondrial respiration, but can upregulate glycolysis to meet ATP demands, and fibroblasts isolated from sporadic ALS patients show an increased dependence on glycolysis with age (Allen *et al.*, 2014, 2015). Similar glycolytic changes have been observed in NSC34 cell models of ALS using a metabolomic approach (Valbuena *et al.*, 2016). This indicates an inherent metabolic flexibility that, at least from a bioenergetic perspective, may allow the cells to initially adapt to the underlying disease pathophysiology.

The additional advantage of fibroblasts is that they can be reprogrammed into induced neuronal progenitor cells (iNPCs), which can in turn be differentiated into induced astrocytes. ALS patient-derived induced astrocytes, have been shown to cause increased toxicity to wild-type motor neurons when grown in co-culture compared to control derived induced astrocytes (Meyer *et al.*, 2014). Astrocytes, along with oligodendrocytes, have a key role in the metabolic support of motor neurons and have a significant influence on the rate of disease progression (Pellerin and Magistretti, 1994; Lin *et al.*, 1998; Clement *et al.*, 2003; Yamanaka *et al.*, 2008; Ferraiuolo *et al.*, 2011*a*, *b*, 2016; Tefera and Borges, 2016). Given that clinical evidence supports a negative impact of dysfunctional energy metabolism on the disease progression in ALS (Desport *et al.*, 2005; Dupuis *et al.*, 2011), it is vital to understand how ALS affects astrocyte metabolic processes including metabolic flexibility (the ability of a cell to mobilize and use alternative substrates to meet energy demands). This understanding would offer the potential for the pathways in question to be manipulated with strategies to improve bioenergetic output and increase the metabolic support for motor neurons.

A recent publication from our laboratory used a novel approach to identify *C9orf72*-dependent metabolic dysfunctional pathways (Allen *et al.*, 2019). Using both patient derived fibroblasts and reprogrammed human induced astrocytes, we used a phenotypic metabolic profiling approach (Bochner *et al.*, 2011; Boccuto *et al.*, 2013) to identify deficient metabolic pathways. This technology enables the comparison of normal versus disease model cells by simultaneously comparing the rates of energy production from 91 potential energy substrates. This approach had not been used previously in the ALS field and allowed a non-biased metabolic screen to be performed on *C9orf72* cell models to identify novel dysfunctional metabolic pathways. We identified an adenosine metabolic dysfunction in *C9orf72* cell models caused by loss of adenosine deaminase, which was also present in sporadic induced astrocytes. Bypassing the defect with inosine supplementation *in vitro* increased induced astrocyte bioenergetic flux and ATP levels, as well as reducing astrocyte-mediated neuronal toxicity. In this present study, we have assessed the ability of our novel metabolic profiling approach to distinguish between controls and *C9orf72* ALS cases using principal component analysis (PCA). We have used our novel screening approach to show that induced astrocytes from *C9orf72* ALS cases display a significant reduction in metabolic flexibility and we have interrogated the pathways involved in this loss of flexibility. We present for the first time alterations in glycogen metabolism, fructose metabolism, methylglyoxal detoxification and mitochondrial substrate transport, which all could contribute to a loss of metabolic flexibility in *C9orf72* astrocytes, making them vulnerable to starvation-induced cell stress under times of bioenergetic deficit.

## Materials and methods

All chemicals are from Sigma unless stated otherwise.

### Human biosamples

Experiments were carried out using samples obtained from six *C9orf72* HRE-positive ALS cases, eight SALS cases and eight matched controls (Supplementary Table 1). The average age at time of skin biopsy in ALS cases (seven females, seven males) was 54 [standard deviation (SD)±12.0] years and 55.1 (±8.8) years in controls (six females, three males). The average disease duration of the ALS cases was 40.0 (±21.7) months. The average age at time of skin biopsy in sporadic Parkinson’s disease cases (two females) was 53.5 (±3.5) years and 53.3 (±3.9) years in age matched controls (three males, one female).

### Ethical approval

Informed consent was obtained from all human subjects before skin sample collection (Study number STH16573 and STH16350, Research Ethics Committee reference 12/YH/0330). All applicable international, national, and/or institutional guidelines for the care and use of animals were followed.

### Human fibroblast cultures

Sterile skin biopsies were obtained from the forearm of subjects after informed consent, in accordance with guidelines set by the local ethics committee. Fibroblast cell cultures were established at the Sheffield Institute for Translational Neuroscience. Monolayers of primary fibroblast cell cultures were routinely maintained in T75 flasks in fibroblast cell culture medium (Lonza) supplemented with 10% foetal calf serum (Labtech), 2 mM glutamine (Lonza BE17–605 E), 50 µg/ml uridine (Sigma U3003), vitamins (Lonza 13–607 C 1/100 dilution), amino acids (Lonza BE13–114E 1/100 dilution), 1 mM sodium pyruvate (Lonza BE13–115E) in humid incubators at 37°C supplemented with 5% CO_2_.

### Human induced astrocyte or neuron culture

Fibroblasts were differentiated as previously described (Meyer *et al.*, 2014). INPCs were cultured in Dulbecco’s modified Eagle medium (DMEM) containing 1% N2 supplement (Life Technologies), 1% B27 supplement, 20 ng/ml fibroblast growth factor-2 (Preprotech). INPCs were differentiated into induced astrocytes on 10 cm dishes coated with fibronectin (5 µg/ml, Millipore) by changing the media to DMEM with 10% foetal bovine serum (FBS) and 0.3% N2 and allowed to differentiate for 7 days. Induced astrocyte markers of differentiation were assessed by immunocytochemistry, PCR as described previously (Meyer *et al.*, 2014; Ferraiuolo *et al.*, 2016) and western blot analysis. Briefly for immunocytochemistry, 10 000 induced astrocytes were plated in 96-well plates at Day 6 of differentiation and fixed 24 h after seeding with 4% paraformaldehyde (PFA) for 10 min and washed three times with phosphate-buffered saline (PBS) before the blocking solution consisting of PBS with 5% horse serum, 0.05% Triton™ X-100, was applied for 1 h. All primary antibodies were diluted in blocking solution and their dilution and provider are listed as follows: chicken vimentin 1:1000 (Millipore, AB5733), rabbit GFAP 1:1000 (Dako, Z0334), rabbit CD44 1:200 (Abcam, ab157107) and goat EAAT2 1:100 (SantaCruz, sc-7760). Incubation of the primary antibody was performed overnight at 4°C. The next day, cells were washed three times in PBS before the secondary antibodies at 1:1000 in blocking solution was applied for 1 h at room temperature. Secondary antibodies used included, Alexa Fluor® 586 goat α-rabbit IgG (H + L) (Invitrogen, A11011), Alexa Fluor® 488 goat α-rabbit IgG (H + L) (Invitrogen, A11006) and Alexa Fluor® 488 goat α-chicken IgG (H + L) (Invitrogen, A11039). Hoechst (Hoechst 33342, Trihydrochloride, Trihydrate, Life Technologies) diluted 1:6000 was added for 5 min to visualize the nucleus. Cells were then washed twice with PBS and imaged using the Opera Phenix™ high-content imager (PerkinElmer). For quantification, all data were uploaded into Columbus (PerkinElmer), nuclei were detected using Hoechst 33342 with a size threshold of 30 µm^2^. Cytoplasmic staining was measured by Alexa 568/488 detection, all intensity properties were calculated as mean intensity per cell. Ninety-ninth background intensity was calculated using the secondary antibody only for each fluorophore and added as a threshold. Percentage of cells with positive expression was calculated using the following formula: positive cells / total cell × 100. Typically, all induced astrocytes showed 96–99% expression of all markers. For differentiation into neurons, iNPCs were plated in a fibronectin-coated 6-well plate and grown to 70–80% confluence after which iNPC medium was removed and replaced with neuron differentiation medium (DMEM/F-12 supplemented with 1% N2 and 2% B27). On Day 1 post-differentiation, the cells were treated with 2.5 μM DAPT (Tocris) to promote differentiation toward a neuronal lineage. On Day 3, the medium was supplemented with 1 μM retinoic acid (Sigma), 0.5 μM smoothened agonist (SAG) (Millipore) and 2.5 μM forskolin (Sigma) for an additional 7 days.

### Phenotype microarray analysis

All assays were performed as described in detail previously (Allen *et al.*, 2019). Briefly, on Day 1, 96-well phenotype microarray plates had 30 µl of IFM-1 (Biolog) containing 10% dialysed FBS and 0.3 mM glutamine added, and were then incubated overnight at 37°C/5% CO_2_. On Day 2, the IFM-1 fluid, now containing the different metabolites, was transferred to the corresponding wells on the fibronectin-coated plates (0.25 µg/ml in PBS). Twenty microlitres of fibroblasts at 800 000 cells/ml, or 20 µl of astrocytes at 500 000/ml were transferred to each well of the substrate plate and incubated at 37°C/5% CO_2_ for 40 or 24 h, respectively. After the stated incubation time, 10 µl of redox dye mix MA (Biolog) was added to each well and the plates sealed with sterile SealPlate® film to stop gas transfer. Dye colour change was measured using a BMG Omega Pherastar at 590/790 nM for fibroblasts or in an OmniLog™ Phenotype Microarray system at 37°C. All results were normalized to cell number by addition of CyQUANT® (Invitrogen) to each well as per the manufacturer’s instructions and fluorescence was measured using a BMG Omega FLUOstar®. All astrocyte and fibroblast cases were analysed in triplicate. All assay data had background values removed and were normalized to cell number using Microsoft Excel. Subsequently, PCA plots were generated using Qlucore Omics Explorer 3.0, with time point eliminated as a factor and *P* < 0.05 taken as significant. Qlucore calculates the eigenvectors (principal components), which determine the directions of a feature in space, with the eigenvalues determining the magnitude of separation and the variation of the data along axes. Qlucore orders the principal components based on the amount of the total variance captured by each component, considering all variables or samples.

### Cell survival analysis

Cell survival data were assessed from the CyQUANT readings taken at the end of each screen and normalizing the specific substrate in question to the positive glucose controls as 100%, using following the equation:

(1) (CyQUANT value of substrate in question)/(average CyQUANT value of glucose wells)×100

### Metabolic flexibility analysis

Metabolic flexibility for each cell line was calculated by counting any energy substrates used on the screening plate that produced NADH within 80% of the NADH production of the glucose control. Percent of glucose control was calculated using the following equation:

(2) (NADH production of energy substrate)/(glucose control NADH production)×100

Glucose control NADH production was equal to the average of the three glucose control wells (B4–B6) on each plate. Only values within 80% of the glucose control were considered as 20% NADH production was three times the mean standard deviation (SD) of the background values (A2–A3) on each plate. Kruskal-Wallis non-parametric analysis was performed on the values calculated from each plate.

### Mitochondrial function assays using MicroPlate™ substrate plates

For the MicroPlate™ substrate (MFS) assays induced astrocytes were harvested as previously described and resuspended at 666 666 cells/ml in 1 × Biolog cell suspension medium (MAS). Thirty microlitres of cell suspension was added to 15 μl 2 × MAS Buffer (Biolog), 2.5 μl 1.8 mg/ml saponin (Sigma) 2.5 μl water and 10 μl Dye MC (Biolog). This 60 -μl solution was added to each well of an MFS assay plate (Supplementary Table 2), which contains 31 metabolites in triplicate precoated and dried into the wells (rows A–B cytoplasmic, rows C–H mitochondrial) plus three negative wells (no metabolite). The plates were sealed and then incubated in an OmniLog™ phenotypic microarray system for 6 h for kinetic analysis. For the mitochondrial substrate analysis, two-way ANOVA with Sidak post-test correction for multiple comparisons were performed at every time point. Initial rate analysis, as well as area under the curve analysis, was performed on all the kinetic traces. For all AUC and rate analysis see Supplementary Table 3.

### Western blot analysis

Three independent flasks of astrocytes were grown to 80% confluency and harvested as previously described. The cell pellets were washed in PBS and resuspended on ice in 100 µl lysis buffer [89% radio-immunoprecipitation assay (RIPA) buffer (Sigma), 10% protease inhibitor cocktail (Sigma) and 1% phosphatase inhibitors (Sigma)]. After 30 min, the cells were centrifuged at 13 000 rpm, 4°C for 30 min; the supernatant was collected and retained on ice. Protein content of the supernatant was determined using a Bradford assay as per the manufacturer’s instructions. All protein samples were denatured at 95°C for 5 min in 1 × Laemmli buffer and 20 μg of protein was loaded on 10% SDS polyacrylamide gels and protein electrophoresis was performed using Mini-PROTEAN® Tetra Handcast systems (Bio-Rad). Proteins were resolved and transferred to a polyvinylidene difluoride membrane (PVDF, Millipore) at 250 mA for 1 h. The PVDF membranes were incubated for 1 h with blocking solution containing 5% BSA in Tris-buffered saline with 0.01% Tween (TBST). Afterwards, the membranes were incubated overnight at 4°C with the following primary antibodies at 1:1000 dilution in blocking solution: rabbit actin (Abcam ab8227), rabbit glycogen phosphorylase (Proteintech 12075–1-AP), rabbit phosphoglucomutase (Proteintech 15161–1-AP), rabbit GLO1 (Proteintech 15140-AP), rabbit GLO2 (Proteintech 17196–1-AP) rabbit fructokinase, (Proteintech 15681–1-AP), rabbit aldolase-C (Proteintech 14484–1-AP), mouse pyruvate dehydrogenase (Abcam 110330), rabbit pyruvate dehydrogenase phosphate (Abcam 17746), rabbit vinculin (Abcam 155120), mouse GLUT1 (Proteintech 66290–1-lg), rabbit GLUT5 (Insight Biotechnology ABP 53119), chicken vimentin (Millipore AB5733) and rabbit GFAP (Dako, Z0334). Before detection by chemiluminescence (EZ-ECL HRP kit, Biological Industries) using a G:BOX (Syngene), the membranes underwent six 10-min washes in TBST followed by incubation with secondary anti-rabbit/mouse HRP-linked antibody (1:5000, Cell Signalling Technology) for 1 h. Quantification of protein levels were obtained by densitometry using GeneTools software (version 4.03.05, Syngene) to the loading controls.

### Quantitative reverse-transcription polymerase chain reaction

Three independent differentiations of induced astrocytes had RNA extracted using the RNAeasy® Mini kit (Qiagen) and were DNase treated before being converted to cDNA as described previously (Hautbergue *et al.*, 2017). The sequences of all qPCR primers can be found in the Supplementary material, Note 1. Quantitative RT-PCR reactions were performed in duplicate using the Brilliant III Ultra-Fast SYBR® Green QPCR Master Mix (Agilent Technologies) on a CFX 96™ Real-Time System (Bio-Rad). Quantitative RT-PCR data were analysed using CFX Manager 3.1 (Bio-Rad) and GraphPad Prism.

### Statistical analysis

All percentage data were firstly transformed Y = 1/Y, then subsequently Y = logit(Y). To test if the data produced fell under Gaussian distribution, all data prior to statistical analysis were assessed for normal distribution by the D’Agostino and Pearson, the Sharpiro-Wilk and the Kolmogorov–Smirnov normality tests. If normal, an unpaired *t*-test or one way ANOVA with Bonferroni post-test analysis was performed; if not normal, then a Mann-Whitney or a Kruskal Wallis with Dunn’s post-test analysis was performed. Statistical analysis was performed on GraphPad Prism software (version 6.0, San Diego, CA).

### Data availability

Raw data were generated at the University of Sheffield. Derived data supporting the findings of this study are available from the corresponding author on request.

## Results

### *C9orf72* leads to a drop in metabolic flexibility and reduced cell survival in iNPC-derived induced astrocytes

**Figure 1:**
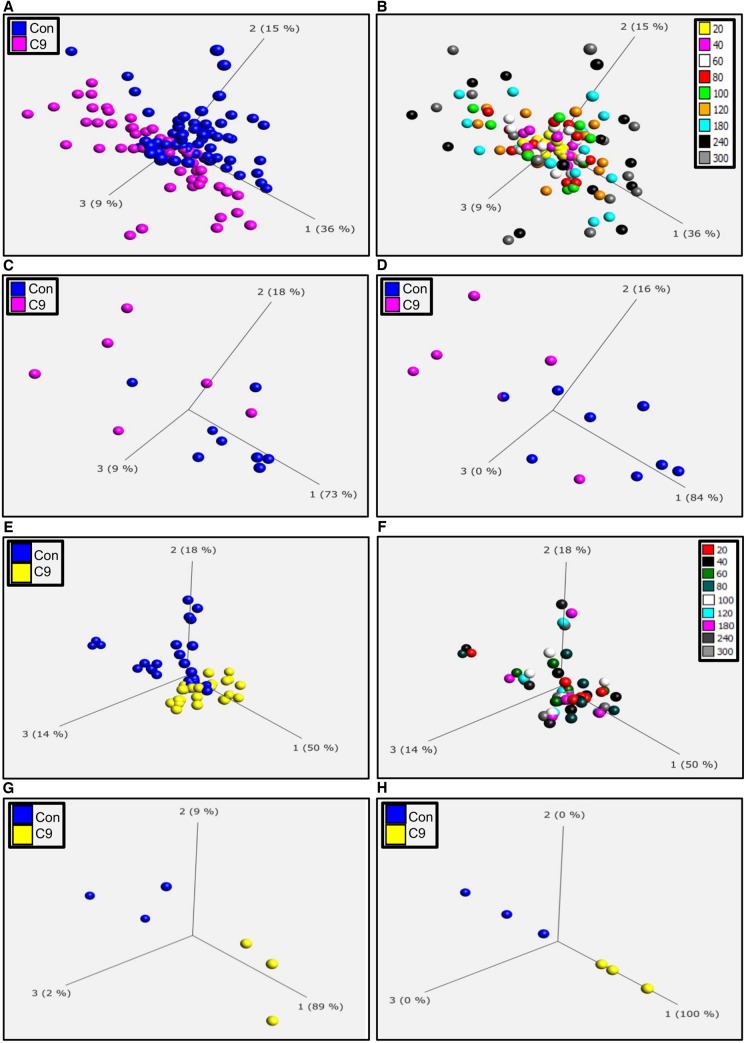
**Cells derived from *C9orf72* fibroblast and induced astrocyte cases have an altered metabolic profile.** (**A**) PCA of control fibroblasts (blue, Con) and *C9orf72* fibroblasts (pink C9) at all time points. (**B**) PCA of fibroblasts coloured for individual time points between 20 and 300 min (**C**) PCA of control fibroblasts (blue, Con) and *C9orf72* patient fibroblasts (pink C9) at 120 min. (**D**) PCA of control fibroblasts (blue, Con) and *C9orf72* fibroblasts (pink C9) at 300 min. (**E**) PCA of control induced astrocytes (blue, Con) and *C9orf72* induced astrocytes (yellow, C9) at all time points. (**F**) PCA of all induced astrocytes coloured for individual time points between 20 and 300 min. (**G**) PCA of control induced astrocytes (blue, Con) and *C9orf72* induced astrocytes (yellow, C9) at 120 min. (**H**) PCA of control induced astrocytes (blue, Con) and *C9orf72* induced astrocytes (yellow, C9) at 300 min. Data are presented as mean of three biological replicates using eight control fibroblasts, six *C9orf72* fibroblasts, three control induced astrocytes and three *C9of72* induced astrocytes. Analysis performed on Qlucore with the *P*-value set to ≤0.05. Q-values were 0.113 for control fibroblasts versus *C9orf72* and 0.073 for control induced astrocytes versus *C9orf72.* Percentage values represent eigenvectors calculated for each analysis. The higher the percentage the greater the confidence of the separation based on the vector.

**Figure 2:**
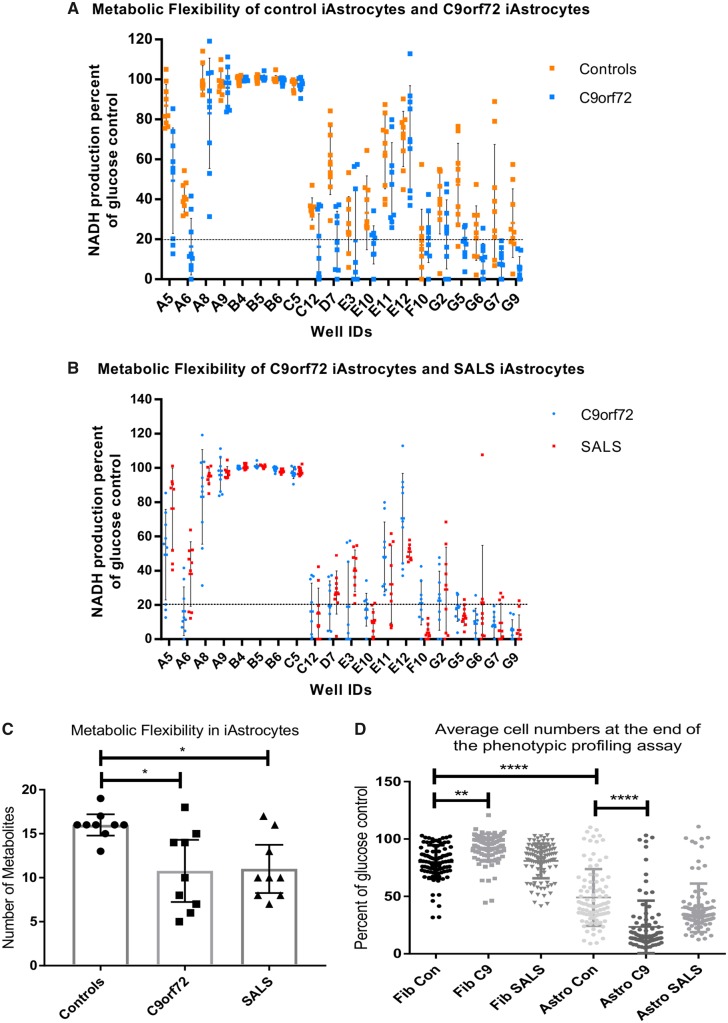
**Metabolic flexibility is reduced in *C9orf72* induced astrocytes.** (**A**) Metabolic flexibility of control induced astrocytes (orange) versus *C9orf72* induced astrocytes (light blue). (**B**) Metabolic flexibility of *C9orf72* (light blue) versus SALS induced astrocytes (red). Data are presented as mean with standard deviation of NADH production as per cent of glucose control (well IDs B4–B6). Metabolic flexibility determined as any energy substrates producing NADH production within 80% of the glucose control. (**C**) Metabolic flexibility in control, *C9orf72* and SALS induced astrocytes. Data are presented as median with 95% confidence intervals followed by one-way ANOVA with Bonferroni post-test analysis. (**D**) Average cell survival in the metabolic profiling assay. Each datapoint represents cell number in one well of one assay for each ALS case or control, showing mean and standard deviation. Data were transformed 1 = 1/Y and 1 = logit (Y) prior to Kruskal Wallis with Dunn’s post-test analysis. The *x*-axis displays well IDs from the metabolic screening plate (PM-M1), which corresponds to the position on the 96-well plate, A4 through to H12, each well contains a unique energy substrate apart from B4–B6, which all contain the positive control glucose. A1–A3 are the negative wells and are not included on the graph. *P ≤ 0.05, **P ≤ 0.01, ****P ≤ 0.0001. Astro/iAstrocytes = induced astrocytes; C9 = *C9orf72*; con = control; Fib = fibroblasts.

Fibroblasts from controls and *C9orf72* cases were reprogrammed into iNPCs and differentiated into induced astrocytes. As described previously (Meyer *et al.*, 2014; Ferraiuolo *et al.*, 2016) both control and *C9orf72* induced astrocytes showed comparable levels of astrocyte markers by immunocytochemistry, PCR and western analysis. There was no significant difference in the levels of the astrocyte markers vimentin, GFAP, CD44, EAAT2, ALDH1L1 and AQP4 in controls and *C9orf72* induced astrocytes (Supplementary Fig. 1A–D), indicating a similar differentiation status between the two groups. Fibroblasts and induced astrocytes were then analysed on phenotypic metabolic array plates (PM-M1, Biolog). This methodology allows an unbiased screen of energy substrates that produce the reduced form of nicotinamide adenine dinucleotides (NADH). Each well of the plate contains a single substrate as an energy source and NADH production is monitored by addition of propriety dyes that are reduced into a coloured product in the presence of NADH. To assess whether the presence of a *C9orf72* expansion altered the metabolome producing a distinct metabolic profile compared to controls, we screened fibroblasts and induced astrocytes from *C9orf72* cases and compared them to healthy controls as described previously (Allen *et al.*, 2019) and then analysed the data using PCA. As a *C9orf72* expansion also accounts for a significant proportion of sporadic ALS cases, we also analysed SALS fibroblasts and induced astrocytes in the same way. During the assay, taking all time points into account, there was significant overlap between the two groups in both fibroblasts and induced astrocytes (Fig. 1A and E). However, we found better separation between the two groups at later time points where the greatest variation in the data were observed (Fig. 1B and F). Therefore, when we focused on individual time points later in the assay (120 min, Fig. 1C and G; and 300 min, Fig. 1D and H), we observed greater separation, especially in induced astrocytes between the *C9orf72* cases and controls. When we performed the same experiment using fibroblasts and induced astrocytes isolated from SALS cases, we observed similar results (Supplementary Fig. 2). This PCA analysis indicated that our phenotypic screening approach could be used to identify distinct metabolic profiles in fibroblasts and induced astrocytes isolated from *C9orf72* and SALS cases as observed previously using classical metabolomic approaches (Rozen *et al.*, 2005; Blasco *et al.*, 2010, 2014, 2017; Kumar *et al.*, 2010). As the *C9orf72* expansion altered the overall metabolic profile, we assessed the metabolic flexibility of the cell models within our assay by determining how many of the specific energy substrates [91 in total including the glucose controls (well ID B4–B6)] the cells could use and to determine whether the presence of the *C9orf72* expansion influenced this flexibility. In addition to glucose, control fibroblasts were able to use 10 of 91 substrates (Supplementary Fig. 3A, black points above dashed line), predominantly using saccharide based metabolites, which are located in well IDs A4 to E4 on the *x*-axis. When reprogrammed into iNPC-derived induced astrocytes, this flexibility increased to 16 of 91 (Supplementary Fig. 3A), with a subtle shift in the profile to the use of nucleoside and carboxylic acid metabolites (well IDs E10 to G10) as well as saccharides. No loss of metabolic flexibility was observed in *C9orf72* or SALS fibroblasts compared to controls (Supplementary Fig. 3B and C). However, when comparing control induced astrocytes with *C9orf72* induced astrocytes, metabolic flexibility dropped from 16 to 10 out of 91 (Fig. 2A and C). A reduction in the overall metabolic flexibility was also observed in SALS induced astrocytes (Fig. 2B and C).

NADH production correlated with cell survival, as typically all wells with energy substrates that were used by the cells showed little loss of cell number (Supplementary Fig. 4). In wells where substrates were not used, increased loss of cell number was observed. The average cell survival across the plate in fibroblasts was high, 80% for controls, 92% for *C9orf72* cases and 80% for SALS cases (Fig. 2D). However, when reprogrammed to induced astrocytes, average control survival dropped to 48% (Fig. 2D and Supplementary Fig. 4A) indicating that metabolic flexibility was more important in induced astrocytes than fibroblasts in terms of cell survival. When *C9orf72* induced astrocytes were analysed, flexibility dropped and with it average cell survival to 23% (Fig. 2D and Supplementary Fig. 4B). In SALS cases, average cell survival was reduced but this did not reach significance because of one line (Case SALS-17) being particularly robust compared to the other SALS cases in terms of metabolic flexibility and cell survival (Fig. 2C, D and Supplementary Fig. 4C). These data indicate that the presence of a *C9orf72* expansion reduces the metabolic flexibility of the induced astrocytes, resulting in sensitivity to starvation induced stress, which may reduce cell numbers. This sensitivity was not observed in fibroblast cell lines.

### *C9orf72* alters saponin sensitivity in induced astrocytes and reduces energy substrate transport

**Figure 3:**
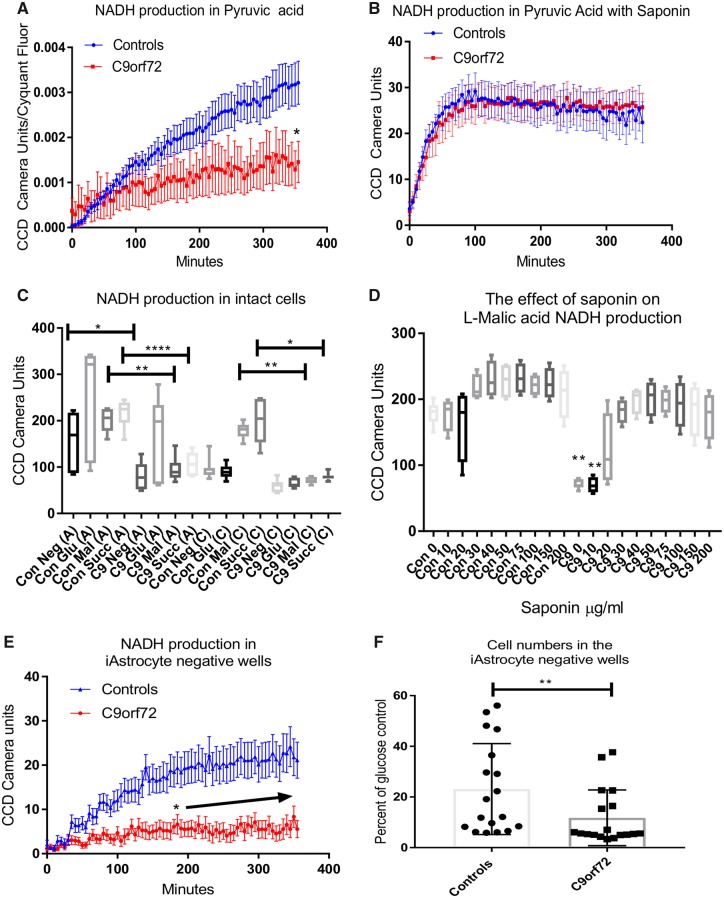
***C9orf72* induced astrocytes have altered saponin sensitivity compared to control induced astrocytes.** (**A**) NADH production in intact induced astrocytes in the presence of pyruvic acid. Data are presented as mean with standard error followed by two-way ANOVA with Sidak post-test analysis in combination with area under the curve (AUC) analysis and initial (0–120 min) linear regression analysis (Supplementary Table 3). (**B**) NADH production in the presence of pyruvic acid with saponin. Data are presented as mean with standard error. (**C**) Induced astrocyte NADH production in the presence of glucose (Glu), l-malic acid (Mal) and succinic acid (Succ). A = Dye A; C = Dye C. Data are presented as mean with standard deviation followed by Kruskal Wallis with Dunn’s post-test analysis. (**D**) The effect of saponin on l-malic acid induced NADH production in induced astrocytes. Data are presented as mean with standard deviation followed by one-way ANOVA with Bonferroni post-test analysis. (**E**) Kinetic NADH production in induced astrocyte negative wells (no energy substrate present). Data presented as mean with standard error followed by two-way ANOVA with Sidak post-test analysis in combination with AUC analysis and initial (0–120 min) linear regression analysis (Supplementary Table 3). (**F**) The effect of starvation on induced astrocyte cell number. Data are presented as mean with standard deviation. Data were transformed 1 = 1/Y and 1 = logit (Y) prior to Mann-Whitney analysis. *P ≤ 0.05, **P ≤ 0.01, ****P ≤ 0.0001. The arrow in **E** denotes consecutive significant time points.

To investigate the loss of metabolic flexibility in the *C9orf72* cases and the starvation-induced toxicity observed further, we used a mitochondrial functional substrate plate recently developed by Biolog Inc. These plates use the same technology as the PM-M1 plates, using a redox dye to assess levels of reduced NADH in the cell. However, the plates are coated with 31 cytosolic and mitochondrial metabolites and cells are permeabilized using saponin to allow transport of the energy substrates into the cell (Supplementary Table 2). This novel assay allowed us to determine whether a reduced ability to transport energy substrates across membranes could contribute to the loss of metabolic flexibility. Seventeen mitochondrial substrates from the original PM-M1 plates were also on the MFS plate, including pyruvic acid, which we observed was hypometabolic in *C9orf72* induced astrocytes in our previous study (Fig. 3A) (Allen *et al.*, 2019). Analysis of NADH production in the presence of pyruvic acid under saponin conditions, showed no significant difference between controls and *C9orf72* induced astrocytes (Fig. 3B). Similar results were observed with alpha ketoglutaric acid, lactic acid, malic acid and succinic acid (Supplementary Fig. 5A–H), which were poorly metabolized by *C9orf72* induced astrocytes in the initial PM-M1 assays (Allen *et al.*, 2019) resulting in starvation-like levels of toxicity (Supplementary Fig. 5J) and therefore at the time were not considered for further analysis. However, in the presence of saponin, NADH production in the controls and the *C9orf72* cases were very similar (Supplementary Fig. 5B, D, F and H), indicating that these substrates were not transported efficiently into the *C9orf72* induced astrocytes in the whole cell assay screen compared to controls. NADH production was observed to a sufficient extent in the presence of seven other metabolites to allow statistical analysis. However, no differences were observed between *C9orf72* and control lines (data not shown). These data indicated that the presence of the *C9orf72* expansion may lead to alterations in the protein/lipid content of the plasma membrane which may affect metabolite transport. To test this hypothesis we performed saponin sensitivity assays in induced astrocytes using the mitochondrial TCA cycle substrates, malic acid and succinic acid with increasing saponin concentrations. As expected, in the absence of saponin both the controls and *C9orf72* ALS cases metabolized glucose using the dye which picks up electrons from NADH in intact cells (dye A; Fig. 3C and Supplementary Fig. 6, column 1). Unlike the *C9orf72* cases, all control lines could metabolize succinic acid and malic acid giving higher NADH production (Fig. 3C and Supplementary Fig. 6). Similar results were observed using the MFS-1 dye (dye C) as a readout (Fig. 3C and Supplementary Fig. 6, columns 2 and 3).

When increasing levels of saponin were added to the cells there was a dose dependent increase in NADH production in the presence of l-malic and succinic acid in *C9orf72* induced astrocytes, which peaked at 50–75 μg/ml (Fig. 3D and Supplementary Figs 5I and 6, columns 3–12). These data suggested that *C9orf72* induced astrocytes, unlike controls, needed the membrane to be more permeabilized (by the presence of an agent such as saponin) to transport mitochondrial metabolites into the mitochondria to be able to use them for energy generation. Taken together, these data suggest the presence of a *C9orf72* expansion may alter membrane transport of energy substrates.

### The *C9orf72* expansion alters glycogen mobilization in induced astrocytes

**Figure 4:**
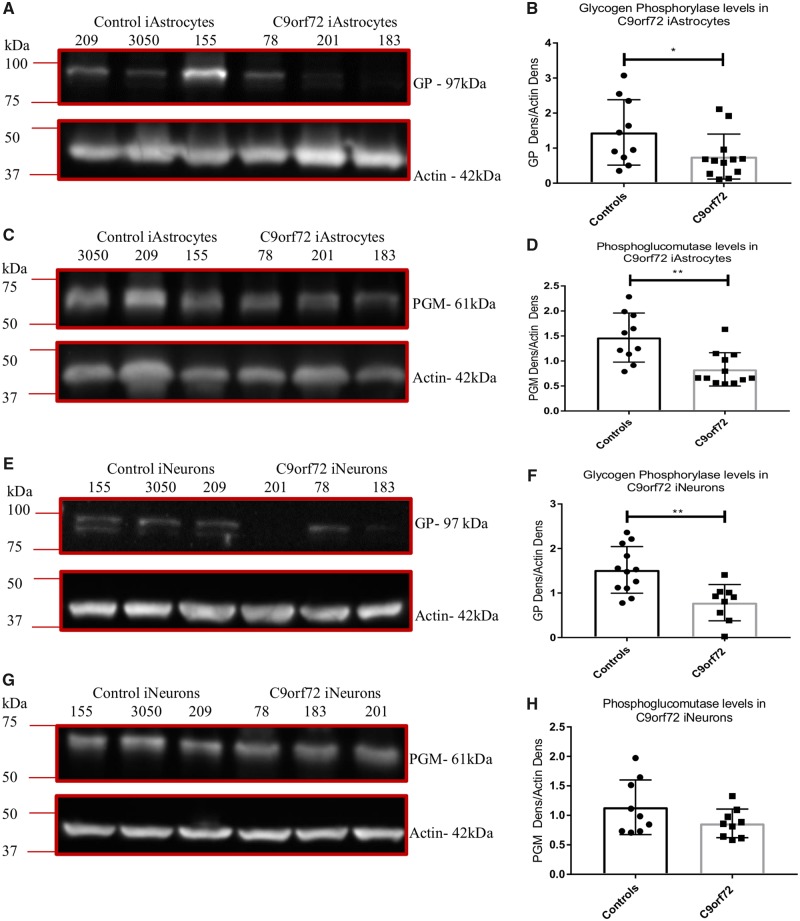
***C9orf72* induced astrocytes and induced neurons have reduced glycogen metabolism enzymes.** (**A** and **B**) Induced astrocyte GP levels. (**C** and **D**) Induced astrocyte PGM levels. (**E** and **F**) Induced neuron GP levels. (**G** and **H**) Induced neuron PGM levels. Densitometry analysis performed by normalizing the protein level of interest to the loading control (actin). Representative western blot of three controls versus three *C9orf72* cases performed *n* = 3/4 before densitometry analysis followed by a Mann-Whitney (**B** and **D**) or an unpaired *t*-test (**F** and **H**). *P ≤ 0.05. iAstrocytes = induced astrocytes; iNeurons = induced neurons.

An additional interesting observation from the saponin assays was that in all the control lines a noticeable colour change was observed in the negative (no substrate) wells (Supplementary Fig. 6, four top wells in column 1) whereas the *C9orf72* cases displayed little colour change. When we quantified this, we saw a significant difference in NADH production between controls and *C9orf72* induced astrocyte lines (Fig. 3C). We also saw similar results in the original screen with a reduction in NADH production in the no substrate wells in *C9orf72* induced astrocytes (Fig. 3E) and concomitantly increased cell death at the end of the assay (Fig. 3F). Under conditions of metabolic starvation, astrocytes rely on the mobilization of internal fuel stores such as glycogen to meet energy demands. Our previous publication (Allen *et al.*, 2019) indicated that *C9orf72* induced astrocytes showed decreased NADH production when glycogen was the sole fuel source. This, coupled with the loss of NADH production under starvation conditions, suggested that the presence of the *C9orf72* expansion may lead to a disruption in glycogen mobilization. To investigate this finding in more detail we measured the levels of glycogen phosphorylase (GP) and phosphoglucomutase (PGM) in *C9orf72* induced astrocytes at the protein and mRNA levels. A significant decrease in both enzymes at the protein level was observed in *C9orf72* ALS lines compared to controls (Fig. 4A–D), similar results were observed at the mRNA level (Supplementary Fig. 6D and E). To determine whether the alteration in glycogen mobilization enzymes was limited to induced astrocytes, we assessed the levels of GP and PGM in induced neurons. The data indicated that GP levels were reduced in *C9orf72* in induced neurons (Fig. 4E and F), whilst PGM levels were reduced but to a level that did not reach significance (Fig. 4G and H).

### The *C9orf72* expansion alters fructose metabolism and impairs methylglyoxal removal enzymes

**Figure 5:**
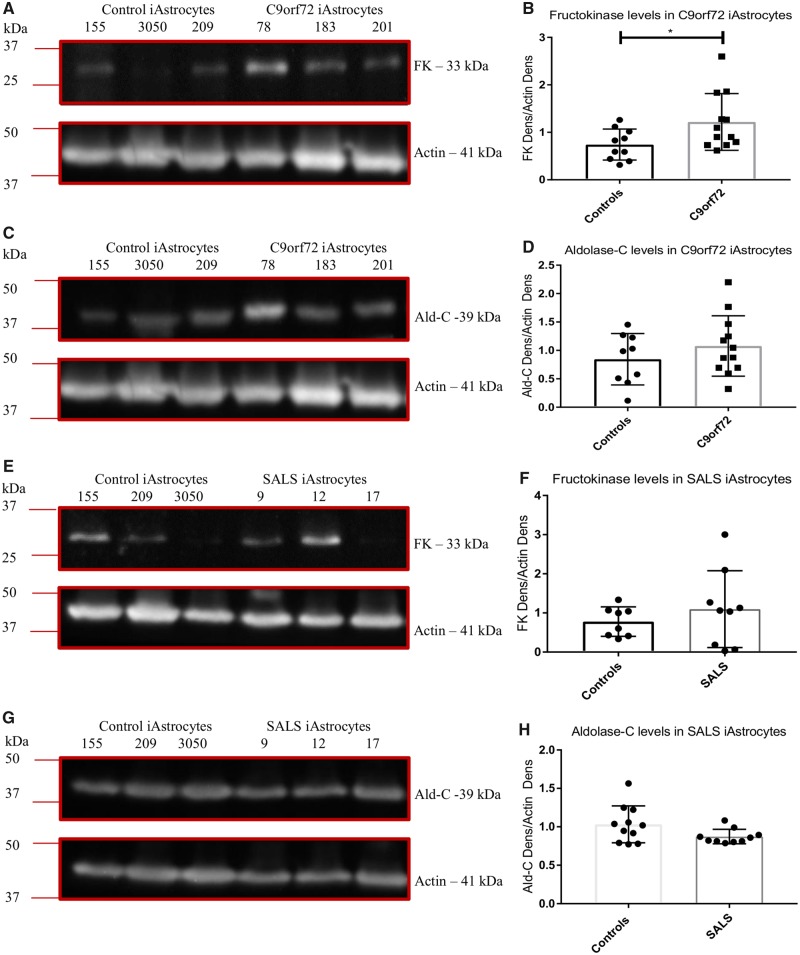
***C9orf72* induced astrocytes have altered levels of fructokinase.** (**A** and **B**) *C9orf72* induced astrocyte fructokinase levels (FK). (**C** and **D**) *C9orf72* induced astrocyte aldolase-C levels (Ald-C). (**E** and **F**) SALS induced astrocyte fructokinase levels. (**G** and **H**) SALS induced astrocyte aldolase-C levels (Ald-C). Densitometry analysis performed by normalizing the protein level of interest to the loading control (actin). Representative western blot of three controls versus three ALS cases performed *n* = 3 before densitometry analysis. Data are presented as mean and standard deviation from three independent biological replicates. All data analysed by an unpaired *t*-test analysis. *P ≤ 0.05. iAstrocytes = induced astrocytes.

As with a glycogen metabolism defect, our previous publication using this screening methodology detected a reduction in NADH production in the presence of fructose, perhaps indicating dysfunction in the fructose metabolism pathway (Allen *et al.*, 2019). To assess this further, we measured the levels of fructokinase and aldolase-C, the enzymes responsible for metabolism of fructose to fructose 1-phosphate and then glyceraldehyde, respectively. Surprisingly we found a modest increase in fructokinase levels in *C9orf72* ALS cases (Fig. 5A and B) although no increase in aldolase-C was observed (Fig. 5C and D). The NADH production step from fructose metabolism occurs downstream of the activity of these two enzymes, when glyceraldehyde 3-phosphate is converted to 1,3-bisphosphoglycerate. If the levels of fructokinase and aldolase-C were unaffected or raised, this suggested a defect further downstream perhaps leading to an increase of glyceraldehyde-3-phosphate (GAP) and dihydroxyacetone phosphate (DHAP). Fructose is a potent glycating agent, leading to toxic advanced glycation end products (AGEs) that can interfere with many cell functions including lipid synthesis, antioxidant defences, and mitochondrial metabolism. Disrupted fructose metabolism leads to the generation of GAP and DHAP, which are both efficient glycating agents and can be converted to dicarbonyls such as glyoxal and methylglyoxal, which in turn leads to more stable AGE production. To combat this, cells have an effective detoxifying mechanism that plays a major role in the cellular defence against glycation and oxidative stress (Thornalley, 1993*a*, *b*; Kalapos, 2008*a*, *b*). The glyoxalase system contains two sequential enzymatic reactions catalysed by glyoxalase-1 (GLO1) and glyoxalase-2 (GLO2), using glutathione as a co-factor. GLO1 converts the hemithioacetal product formed by the reaction of reduced glutathione with methylglyoxal, to *S*-d-lactoylglutathione. This compound is then metabolized by GLO2 to d-lactate, which recycles glutathione. As *S*-d-lactoylglutathione is non-toxic GLO1 activity is crucial for methylglyoxal detoxification and determines the rate of AGE formation (Allaman *et al.*, 2015).

**Figure 6:**
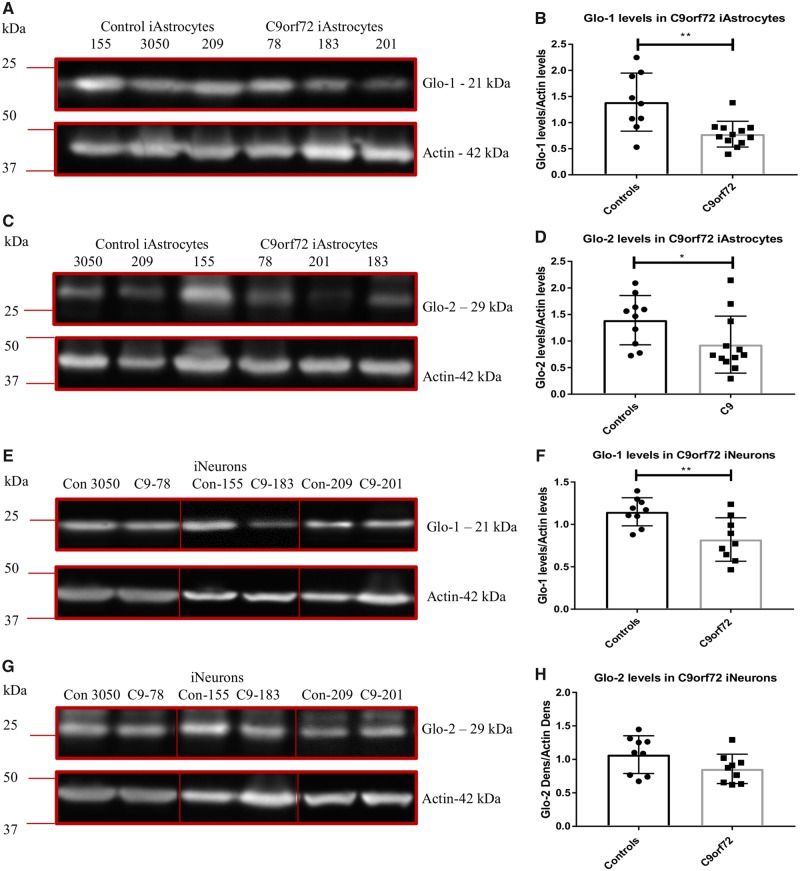
***C9orf72* induced astrocytes and induced neurons have reduced methylglyoxal removal enzymes.** (**A** and **B**) Induced astrocyte GLO1 levels. (**C** and **D**) Induced astrocyte GLO2 levels. (**E** and **F**) Induced neuron GLO1 levels. (**G** and **H**) Induced neuron GLO2 levels. Densitometry analysis performed by normalizing the protein level of interest to the loading control (actin). Representative western blot of three controls versus three *C9orf72* cases performed *n* = 3/4 before densitometry analysis and analysis by an unpaired *t*-test (**B** and **F**) or a Mann-Whitney test (**D**). *P ≤ 0.05, **P ≤ 0.01. Lines on blots indicated cropped images. For full length blots, see Supplementary Fig. 8C. iAstrocytes = induced astrocytes; iNeurons = induced neurons.

To assess whether levels of these detoxifying enzymes were altered in *C9orf72* induced astrocytes, we measured protein levels of GLO1 by western blot analysis (Fig. 6A and B) and mRNA levels by RT-PCR (Supplementary Fig. 7A). We found a significant reduction in the levels of GLO1 in the *C9orf72*-ALS lines compared to controls. To assess whether this was limited to induced astrocytes we measured GLO1 levels in *C9orf72* induced neurons and found a similar reduction (Fig. 6E and F). Although we found a significant reduction of GLO2 at the protein level in *C9orf72* induced astrocytes (Fig. 6C and D) this was not matched at the mRNA level (Supplementary Fig. 7B) or at the protein level in induced neurons (Fig. 6G and H).

**Figure 7:**
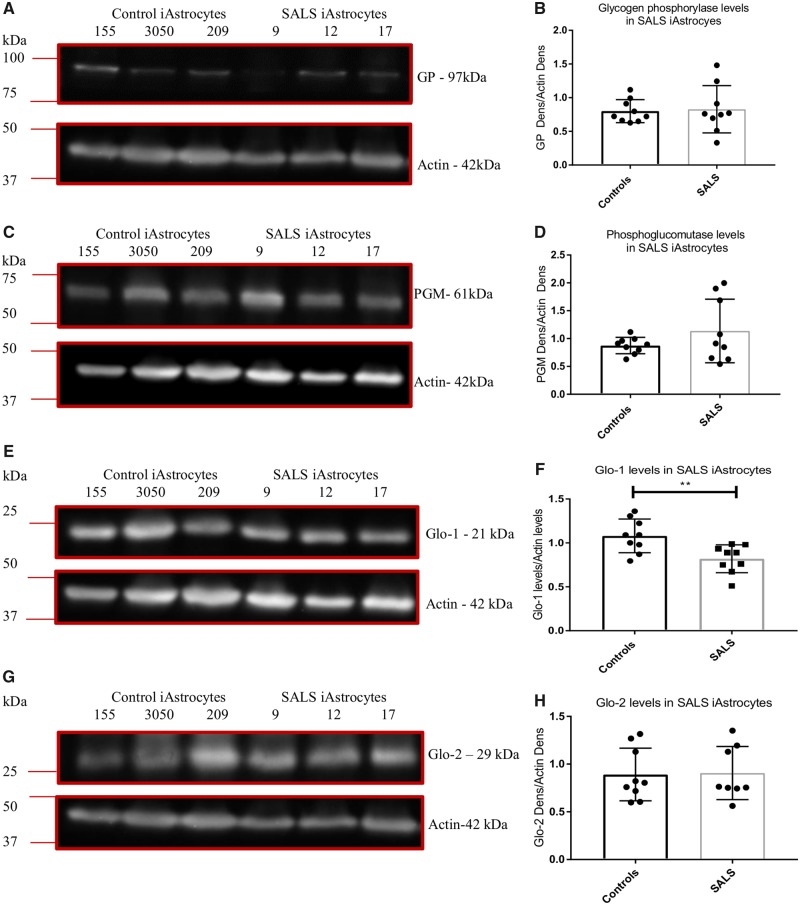
**SALS induced astrocytes have reduced GLO1 levels.** (**A** and **B**) Induced astrocyte GP levels. (**C** and **D**) Induced astrocyte PGM levels. (**E** and **F**) Induced astrocyte GLO1 levels. (**G** and **H**) Induced astrocyte GLO2 levels. Densitometry analysis performed by normalizing the protein level of interest to the loading control (actin). Representative western blot of three controls versus three SALS cases performed *n* = 3 before densitometry analysis followed by an unpaired *t*-tests. **P ≤ 0.01. iAstrocytes = induced astrocytes.

In a recently published paper, we found similar alterations at the protein level of adenosine deaminase in both *C9orf72* and SALS cases (Allen *et al.*, 2019). As a *C9orf72* expansion accounts for a significant proportion of sporadic ALS cases as well as familial cases, we wanted to determine whether these findings were limited to *C9orf72* ALS cases. To assess this, we measured the protein levels of glycogen phosphorylase, PGM, fructokinase, aldolase-C GLO1 and GLO2 in the three SALS induced astrocytes lines used in our previous studies and used to generate PCA plots in this study. Unlike the *C9orf72* induced astrocytes no consistent loss of the glycogen enzymes were observed in SALS induced astrocytes (Fig. 7A–D). However, as evident from the data (Fig. 7A–D), we observed SALS case-specific alterations in the glycogen mobilization enzymes. Case SALS-9 had reduced GP compared to controls (Fig. 7A and lowest three datapoints in Fig. 7B, unpaired *t-*test *P = *0.032) and upregulated PGM (Fig. 7C, highest three datapoints in Fig. 7D, unpaired *t*-test *P = *0.0014), perhaps as a cellular response to GP reduction. Case SALS-17 had no change in GP but showed a mild but significant reduction in PGM levels (unpaired *t*-test *P = *0.041, bottom three datapoints in Fig. 7D).

When we assessed the levels of the fructose metabolism enzymes, the data were very similar to the *C9orf72* induced astrocytes. Two out of three SALS cases (Cases SALS-9 and SALS-12) had raised fructokinase levels but overall no significant increase was observed due to the very low levels of fructokinase in Case SALS-17 (Fig. 5E and F). As with the *C9orf72* induced astrocytes, SALS cases showed no change in aldolase-C levels (Fig. 5G and H). Interestingly, when we measured GLO1 levels in the SALS induced astrocytes, GLO1 was consistently decreased in all cases, (Fig. 7E and F). However, no difference was observed with GLO2 (Fig. 7G and H). Similar results were found at the mRNA level (Supplementary Fig. 7C and D).

To confirm the relevance to ALS and to ascertain whether these alterations were ALS specific, we assessed the levels of our target molecules in sporadic Parkinson’s cases and their age- and sex-matched controls. As with our ALS induced astrocytes, the sporadic Parkinson’s induced astrocytes were differentiated from iNPCs that had been reprogrammed from fibroblasts isolated from sporadic Parkinson’s disease cases (Supplementary Table 1). No significant differences in the enzymes responsible for fructose metabolism were observed between the sporadic Parkinson’s disease cases and their age- and sex-matched controls in either induced astrocytes or fibroblasts (Supplementary Fig. 7E–H). In terms of the glycogen enzymes, as with sporadic ALS cases and unlike our *C9orf72* cases, we found no consistent reduction in both glycogen enzymes in sporadic Parkinson’s disease cases. As with our SALS cases we found cell-specific levels of both targets, sporadic Parkinson’s disease Case OB182, for example, had low levels of GP compared to controls but high levels of PGM (Supplementary Fig. 7E and G), as observed with Case SALS-9, perhaps again as a compensatory mechanism.

However, as with our *C9orf72* and sporadic ALS induced astrocytes, we found a consistent significant loss of GLO1 in the sporadic Parkinson’s disease cases compared to the sporadic Parkinson’s disease age and sex matched controls (Supplementary Fig. 7E and G). These differences seem to be CNS specific as when we tested the original fibroblast lines no differences were observed (Supplementary Fig. 7F and H). This loss of GLO1 in both familial and sporadic ALS samples as well as sporadic Parkinson’s samples may suggest a novel common pathway of dysfunction between the two neurodegenerative disorders. However, because of the low number of patients per group further investigation is warranted.

## Discussion

Our data show that phenotypic metabolic screening can be used to distinguish altered metabolic profiles between controls and patient-derived, CNS-specific, ALS models of disease. We have identified that induced astrocytes derived from *C9orf72* ALS cases and SALS cases have an altered metabolic profile compared to controls and display a loss of metabolic flexibility. Fibroblasts overall showed less separation from controls than induced astrocytes (Fig. 1 and Supplementary Fig. 2) and retained their metabolic flexibility compared to controls. This retained flexibility may be one factor to explain why fibroblasts are able to compensate metabolically in the presence of genetic alterations that are injurious for CNS cells such as astrocytes and neurons. We have previously observed altered metabolic responses in fibroblasts from both SOD1 and sporadic ALS cases (Allen *et al.*, 2014, 2015; Raman *et al.*, 2015), but now, with the adoption of our novel metabolic screening approach, we can conclude with confidence that inherent metabolic flexibility is vital in the CNS and is reduced in ALS patients.

Our unique approach of screening under non-physiological conditions has allowed us to identify key points in the metabolic pathway that are affected in *C9orf72* induced astrocytes, which would not have been observed in physiological (non-stress) conditions, including: adenosine metabolism, fructose metabolism and glycogen metabolism. Under times of cellular stress such as the bioenergetic starvation induced during the screening process, this loss of metabolic flexibility contributed to the cell loss observed in the *C9orf72* induced astrocyte model, which was the result of defects in energy substrate transport, combined with a reduced ability to mobilize intracellular energy stores such as glycogen and adenosine to produce NADH (Figs 3, 4 and Allen *et al.*, 2019).

Glycogen is the main storage form of glucose, mainly localized in astrocytes in the CNS and can be metabolized without ATP consumption (Brown, 2004). Decreased glycogen metabolism, either through reduction of GP and PGM or α-glucosidase (GAA), which has previously been shown to be reduced at the mRNA level in gene expression studies on laser captured motor neurons from ALS patients (Dodge *et al.*, 2013), could lead to glycogen accumulation in the CNS. At times of high-energy demand or bioenergetic stress, decreased mobilization of glycogen in ALS cases may cause decreased glucose levels and therefore lead to a reduction in ATP production. This has been reported in grey matter neurons and glia of ALS cases and also in muscle, with glycogen accumulation and diminished ATP levels observed (Dodge *et al.*, 2013). Laser-captured astrocytes from SOD1^G93A^ mice have demonstrated a >2-fold increase in glycogen synthase mRNA (Ferraiuolo *et al.*, 2011*a*). However, we found no consistent increase of glycogen synthase in *C9orf72* induced astrocytes at the protein level (data not shown). Our data suggest a reduced ability to mobilize glycogen rather than a glycogen storage defect is likely to contribute to starvation-induced toxicity. The glycogen defect was more evident in *C9orf72* induced astrocytes, compared to SALS induced astrocytes, which may indicate a distinct *C9orf72* pathogenic mechanism.

However, SALS cases are heterogeneous and firm conclusions are not yet possible given the relatively small number of SALS cases used in this study. In addition, the fact that glycogen level alterations have been observed in tissue from ALS cases and in SOD1 mice indicates that our findings are relevant to the pathogenesis of at least some additional subtypes of ALS.

Disruption in lysosomal glycogen breakdown, as well as a secondary defect in the fusion between autophagosomes and lysosomes, have been implicated in certain myopathies including Pompe disease. Pompe disease is caused by a mutation in GAA, with deficiencies in both humans and in mouse models leading to accumulation of swollen lysosomes swollen containing glycogen (Smith *et al.*, 1967; Raben *et al.*, 2002, 2009; van der Ploeg and Reuser, 2008). Defects in autophagy are linked with several neurological disorders including Parkinson’s disease and ALS (Harris and Rubinsztein, 2011). *C9orf72* has been shown to play a key role in autophagy initiation, with upregulation of P62 and the LC3 II/I ratio observed in *C9orf72* patient-derived cell models indicating an impairment of autophagy (Webster *et al.*, 2018; Allen *et al.*, 2019). A key role of autophagy is to release glucose, fatty acids and amino acids to maintain cellular functions during stress and starvation. It has been shown in mice, that glycogen-rich fast-twitch muscle fibres induce autophagy to a greater extent than oxidative slow-twitch fibres (Mizushima *et al.*, 2004). Therefore, a link may exist between the glycogen/glucose axis and autophagy regulation in ALS. *C9orf72* autophagy disruption may contribute to the bioenergetic deficit observed in ALS patients not only in the CNS but in muscle. Selective targeting of the glycogen/glucose axis by increasing glycogenolysis could potentially compensate for the bioenergetic/autophagy defect and increase energy production via glycolysis. This is a crucial area of metabolic research in ALS, especially in light of the recent publication suggesting a neuroprotective role of glycolysis in TDP43-linked ALS (Manzo *et al.*, 2019). Further mechanistic studies are planned in our laboratory to uncover the link between *C9orf72* hexanucleotide repeat expansions and glycogen metabolism in ALS.

Substrate mobilization also includes the ability to transport substrates across membranes. We found that *C9orf72* induced astrocytes had significantly reduced ability to metabolize mitochondrial energy substrates in intact cells, (Fig. 3 and Supplementary Fig. 5). This resulted in high levels of toxicity (in the whole cell intact screen), which were not statistically different from the levels of toxicity observed under starvation (no energy substrates) conditions (Supplementary Fig. 5J). This was in contrast to the cytoplasmic saccharide based energy substrates identified, which in general did not show a starvation like toxicity profile, with cell numbers significantly higher than those observed under starved, no energy substrate conditions (Supplementary Fig. 5K). These data indicate that the defects observed in the cytoplasmic saccharide based energy substrates described in this study and our previously published study (Allen *et al.*, 2019) were not due to loss of energy substrate transport and were due to alterations in the intracellular enzymes responsible for energy metabolism in these pathways, which we have identified. This is in contrast to pyruvic acid for example, where we saw no difference in the activation state of pyruvate dehydrogenase in the *C9orf72* induced astrocytes compared to controls (Supplementary Fig. 8A and B). This was confirmed by measuring the levels of the ubiquitously expressed glucose transporter 1 (GLUT1), which is responsible for glucose uptake in astrocytes and glucose transporter 5 (GLUT5), which is responsible for fructose uptake (Jurcovicova, 2014). No difference was found between *C9orf72* induced astrocytes and controls in GLUT1 levels indicating that the *C9orf72* induced astrocytes were not starved of saccharide-based cytoplasmic energy substrates (Supplementary Fig. 8D–G). Similar results were found with GLUT5 with or without fructose supplementation, indicating that the fructose metabolism defect observed was not due to a loss of fructose transport or a defect in transporter upregulation in the *C9orf72* induced astrocytes.

Pyruvic acid and lactic acid are transported across cell membranes, including both the plasma and mitochondrial membranes, by monocarboxylate transporters (Halestrap, 2012*a*, *b*). Altered gene expression of monocarboxylate transporter 4, (*SLC16A4*) has been found in the spinal cord of hSOD1^G93A^ mice (Ferraiuolo *et al.*, 2011*a*) and the oligodendrocytic lactate transporter monocarboxylate transporter 1 (*SLC16A1*) was decreased in the spinal cords of ALS cases and SOD1^G93A^ mice (Lee *et al.*, 2012*b*; Philips *et al.*, 2013). The Krebs cycle intermediates that we found were affected, can be taken up from blood by the SLC13 transporters NaDC1, NaDC3 and NaCT. NaDC3 has been shown to translocate alpha-ketoglutaric acid (Kaufhold *et al.*, 2011), but at this point this enzyme has not been linked to ALS.

In saponin-permeabilized cells no significant NADH production differences were observed between controls and *C9orf72* induced astrocytes in the presence of pyruvic acid, lactic acid, succinic acid and l-malic acid. Furthermore, *C9orf72* induced astrocytes displayed altered saponin sensitivity compared to controls (Fig. 3). As saponin functions mainly by solubilizing cholesterol, forming a complex and leaving the membrane intact (Jacob *et al.*, 1991; Bottger and Melzig, 2013), this may suggest a potential alteration in the cholesterol content in induced astrocyte membranes from *C9orf72* ALS cases, which may influence the transport of metabolites in the cell, rather than an enzyme defect. Altered membrane cholesterol content has previously been observed in ALS cases and models of disease (Cutler *et al.*, 2002; Baker *et al.*, 2015). Conversely, a more recent study found no cholesterol content differences in cortical neuron cultures from wild-type and G93A SOD1 mice, but did identify an altered susceptibility to cholesterol depletion in terms of loss of NMDA receptor evoked currents (Antonini *et al.*, 2018). Cholesterol content has been shown to influence the physical properties of membranes including fluidity and rigidity, which may affect the level of both membrane receptor expression internalization, and transporter activity (Yeagle *et al.*, 1988; Ikonen, 2008; Jaipuria *et al.*, 2018; Zhang *et al.*, 2019). Therefore, in-depth analysis of metabolic substrate transporters within both the plasma and mitochondrial membranes and analysis of cholesterol content has the potential to highlight either chemical or genetic manipulation strategies, which may contribute to enhancing astrocyte metabolic flexibility and increasing support to motor neurons. Intriguingly, the defects in mitochondrial energy substrate usage identified in the *C9orf72* induced astrocytes may account for the subtle shift towards a more glycolytic ATP production state in the *C9orf72* induced astrocytes, described in our previous manuscript (Allen *et al.*, 2019).

In addition to glycogen, we have identified a defect in fructose metabolism that may be linked to alterations in the methyglyoxal removal pathway. Fructose is increasingly prevalent in the human diet as, along with glucose, it is a component of sucrose, which is used as an artificial sweetener (Aragno and Mastrocola, 2017). A high-fructose diet has been associated with insulin resistance and dyslipidaemia, as well as causing inflammation and reduction of peroxisome proliferator-activated receptor alpha (PGC1A) in animal models (Livesey and Taylor, 2008; Miller and Adeli, 2008; Mock *et al.*, 2017). As fructose enters glycolysis at DHAP and GAP, it bypasses phosphofructokinase, the glycolytic rate-limiting enzyme and the need for insulin. Enhanced exposure to fructose, combined with defects in the fructose metabolic pathway, may lead to enhanced protein glycation through accumulation of DHAP and GAP as well as enhanced methylglyoxal production (Hamada *et al.*, 1996). Accumulation of toxic AGEs has been linked to mitochondrial dysfunction and oxidative stress in several neurodegenerative diseases including Parkinson’s disease (Seneff *et al.*, 2011; Li *et al.*, 2012; Hipkiss, 2014; Mastrocola *et al.*, 2016) and in ALS (Kaufmann *et al.*, 2004; Juranek *et al.*, 2015, 2016; Kim *et al.*, 2018). Currently there are limited data focusing on methylglyoxal and its removal in ALS (Sirangelo *et al.*, 2016) and to the best of our knowledge, this is the first study to suggest that GLO1 levels are altered in ALS patient-derived cell models. However, the fact that we found similar alterations in the GLO1 levels in SALS induced astrocytes and sporadic Parkinson’s disease cases suggests that an important novel common mechanism of dysfunction may exist between ALS and Parkinson’s disease. Recently, the protein sensor DJ-1, implicated in Parkinson’s disease (Bonifati *et al.*, 2003*a*, *b*), was identified as having glyoxalase activity (Lee *et al.*, 2012*a*) with methylglyoxal moieties leading to its downregulation and glycation of α-synuclein (Sharma *et al.*, 2019). This indicates that as with ALS, effective removal of glycation agents such as methylglyoxal in Parkinson’s disease is crucial in protecting against oxidative stress and mitochondrial dysfunction in the CNS, which play key pathogenic roles in both diseases (Barber and Shaw, 2010; Toyoda *et al.*, 2014; Smith *et al.*, 2017; Macdonald *et al.*, 2018; Mortiboys *et al.*, 2018; Dolgacheva *et al.*, 2019).

Our findings that GLO1 levels are reduced in induced astrocytes derived from ALS and Parkinson’s patients are interesting especially in light of the recent findings that Nrf-2, a key regulator of oxidative stress that is normally sequestered and inactivated by the Kelch-like ECH associated protein 1 (KEAP1), is released and activated by accumulation of methylglyoxal (Bollong *et al.*, 2018). This metabolically regulated communication between metabolism and the oxidative stress response via methylglyoxal is a key cellular protection pathway, which would be vital in ALS and Parkinson’s disease where metabolic dysfunction and oxidative stress are important players in disease pathophysiology. Further studies are planned in our laboratory to investigate the effect of loss of GLO1 on this cellular protection axis.

## Supplementary Material

awz302_Supplementary_Materials

## Abbreviations

GP: glycogen phosphorylase
iNPC: induced neuronal progenitor cells
PCA: principal component analysis
PGM: phosphoglucomutase
(S)ALS: (sporadic) amyotrophic lateral sclerosis
