# Supplementary material for: C9orf72 expansion within astrocytes reduces metabolic flexibility in amyotrophic lateral sclerosis
Source: Brain. 2019 Oct 24;142(12):3771–90. doi: 10.1093/brain/awz302 (PMC6906594; doi:10.1093/brain/awz302)
Supplement: awz302_Supplementary_Materials [file awz302_supplementary_materials.zip › awz302-suppl_data/awz302_Supplementary Note and Supplementary Figs_FINAL.pdf]

Supplementary Note 1

Human GP:

Forward 5’-CATGTTCGATGTGCATGTGA-3’

Reverse 5’-TTTCAACCTGTCACCCACAA-3’

Human PGM:

Forward 5’-TCTGACAGCCAGTCACAACC-3’

Reverse 5’-GGCGATCCTTTTGATASGCCTCC-3’

Human Glo-1:

Forward 5’-AATTGGGGCACTGAAGATGA-3’

Reverse 5’-CCAGGCCTTTCATTTTACCA-3’

Human Glo-2:

Forward 5’-GGTCTCTGAACGTCAAGTGCCT-3’

Reverse 5’-CCAGCCACAAACAAGGTGTCAC-3’

Human CD44

Forward 5’- AATGGAGCTGTGGAGGACAG -3’

Reverse 5’- ACTGGTCTGGAGTTTCTGACG -3’

Human Aqp4

Forward 5’- GCACCAGGAAGATCAGCATCG -3’

Reverse 5’- GAGACCATGACCAGCGGTAAG -3’

Human Aldh1L1

Forward 5’- TCACAGAAGTCTAACCTGCC -3’

Reverse 5’- AGTGACGGGTGATAGATGAT -3’

Human U1snRNA

Forward: 5’-CCATGATCACGAAGGTGGTT-3’

Reverse: 5’-ATGCAGTCGAGTTTCCCACA-3’

Human RLP13A

Forward: 5’- CAAGCGGATGAACACCAACC -3’

Reverse: 5’- TTTTGTGGGGCAGCATACCT -3’

Supplementary Figure 1. Differentiation characterisation of control and *C9orf72* iAstrocytes

(A) Representative immunofluorescence images of expression of the astrocytic markers vimentin, CD44, GFAP and EAAT in control and *C9orf72* iAstrocytes. Hoechst (blue) was used to visualise nuclei (Scale bar: 20/50µM). **(B)** Quantitative analysis of the immunocytochemistry images. Data presented as mean with standard deviation. **(C)** mRNA of the differentiation targets Aldh1L1, Aqp4 and CD44 normalised to RLP13A in iAstrocytes. Data presented as mean with standard deviation. **(D)** Representative western blot showing levels of vimentin and GFAP in control and *C9orf72* iAstrocytes.

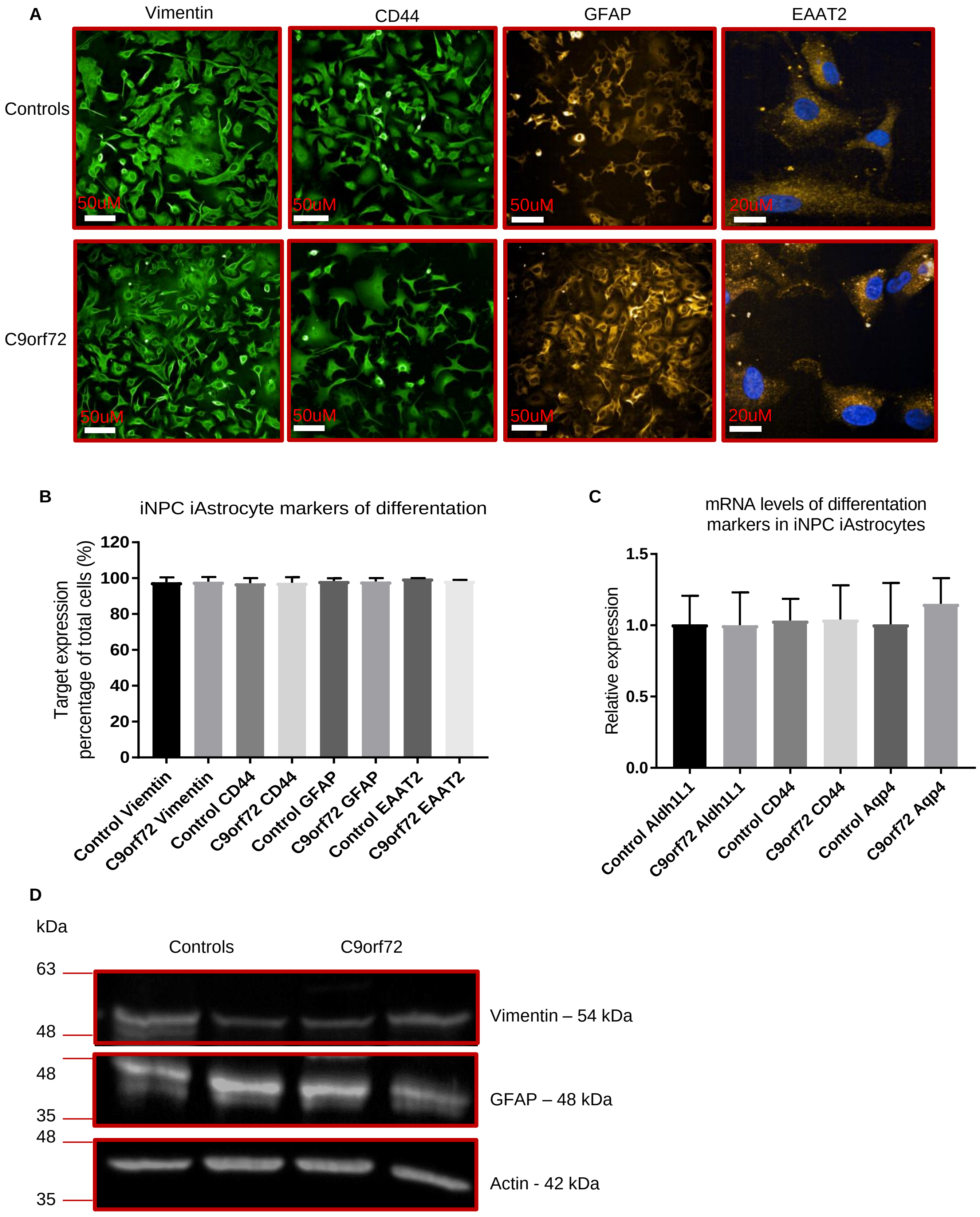

A
Vimentin
CD44
GFAP
EAAT2
Controls
C9orf72
50uM
20uM
B
iNPC iAstrocyte markers of differentation
Target expression percentage of total cells (%)
0
20
40
60
80
100
120
Control Viemtin
C9orf72 Vimentin
Control CD44
C9orf72 CD44
Control GFAP
C9orf72 GFAP
Control EAAT2
C9orf72 EAAT2
C
mRNA levels of differentation markers in iNPC iAstrocytes
Relative expression
0.0
0.5
1.0
1.5
Control Aldh1L1
C9orf72 Aldh1L1
Control CD44
C9orf72 CD44
Control Aqp4
C9orf72 Aqp4
D
kDa
Controls
C9orf72
63
48
35
Vimentin – 54 kDa
GFAP – 48 kDa
Actin - 42 kDa

Supplementary Figure 2. Cells derived from SALS fibroblast and iAstrocyte cases have an altered metabolic profile

(A) Principle component analysis of control fibroblasts (blue, Con) and SALS fibroblasts (yellow, SALS) at all-time points. (**B**) Principle component analysis of fibroblasts coloured for individual time points between 20 and 300 minutes (**C**) Principle component analysis of control fibroblasts (blue, Con) and SALS fibroblasts (yellow, SALS) at 120 minutes (**D**) Principle component analysis of control fibroblasts (blue, Con) and SALS fibroblasts (yellow, SALS) at 300 minutes. (**E**) Principle component analysis of control iAstrocytes (blue, Con) and SALS iAstrocytes (yellow, SALS) at all-time points. (**F**) Principle component analysis of all iAstrocytes coloured for individual time points between 20 and 300 minutes **(G)** Principle component analysis of control iAstrocytes (blue, Con) and SALS iAstrocytes (yellow, SALS) at 120 minutes. **(H)** Principle component analysis of control iAstrocytes (blue, Con) and SALS iAstrocytes (yellow, SALS) at 300 minutes. Data presented as mean of three biological replicates using 7 control fibroblasts, 5 SALS fibroblasts, 3 control iAstrocytes and 3 SALS iAstrocytes n = 3. Analysis performed on Qlucore with the P value set to ≤ 0.05. Q values were 0.065 for control fibroblasts vs SALS and 0.077 for control iAstrocytes vs SALS. Percentage values represent eigenvectors calculated for each analysis. The higher the percentage the greater the confidence of the separation based on the vector.

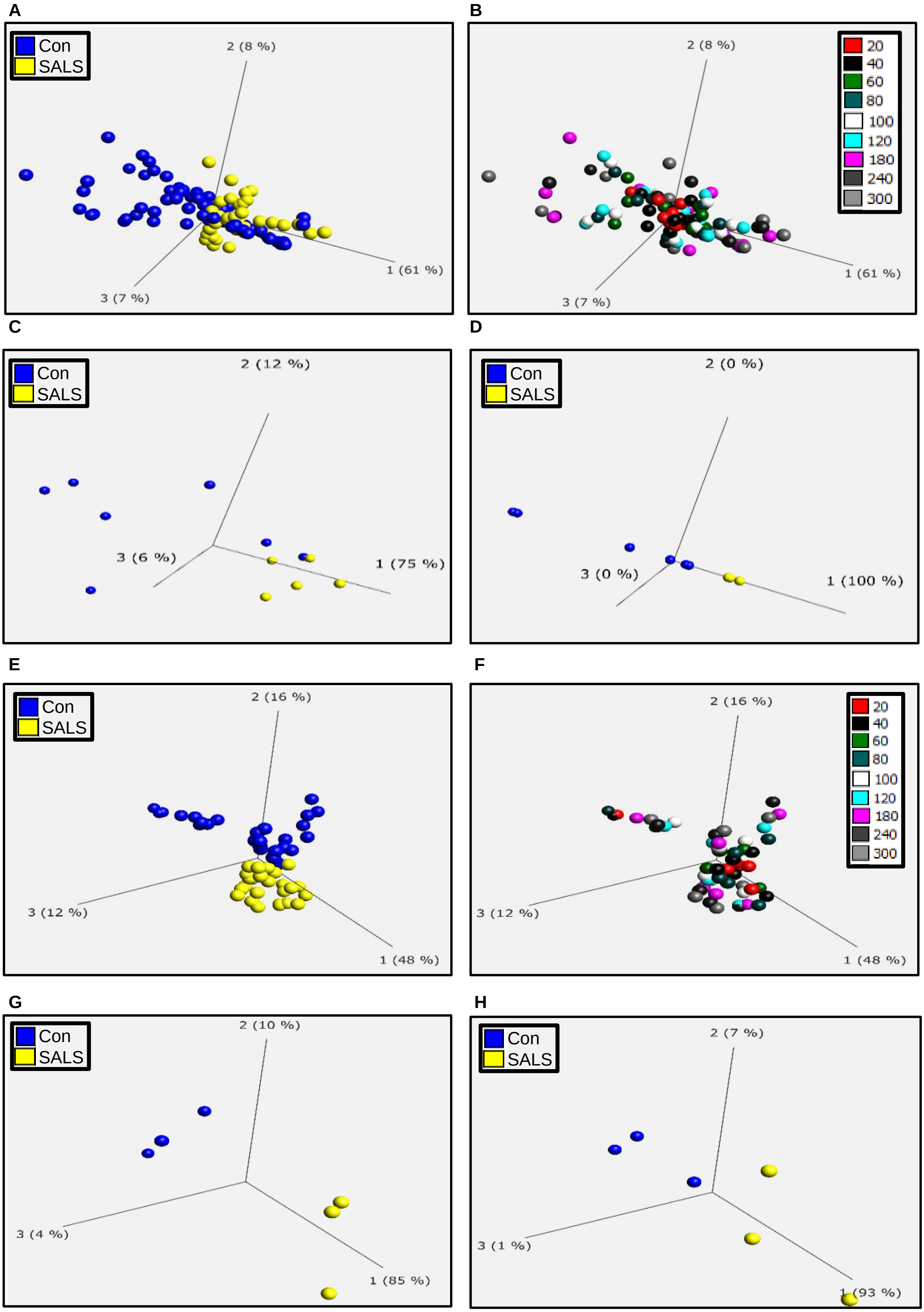

A
Con
SALS
2 (8 %)
1 (61 %)
3 (7 %)
B
2 (8 %)
20
40
60
80
100
120
180
240
300
1 (61 %)
3 (7 %)
C
Con
SALS
2 (12 %)
3 (6 %)
1 (75 %)
D
Con
SALS
2 (0 %)
3 (0 %)
1 (100 %)
E
Con
SALS
2 (16 %)
3 (12 %)
1 (48 %)
F
2 (16 %)
20
40
60
80
100
120
180
240
300
3 (12 %)
1 (48 %)
G
Con
SALS
2 (10 %)
3 (4 %)
1 (85 %)
H
Con
SALS
2 (7 %)
3 (1 %)
1 (93 %)

Supplementary Figure 3. **Metabolic flexibility in fibroblasts from controls, *C9orf72* and SALS cases**

(**A**) Metabolic flexibility of control fibroblasts (black) vs control iAstrocytes (orange). (**B**) Metabolic flexibility of control fibroblasts (black) vs *C9orf72* fibroblasts (red). (**C**) Metabolic flexibility of control fibroblasts (black) vs SALS fibroblasts (blue). Data presented as mean with standard deviation of NADH production as percent of glucose control. Metabolic flexibility determined as any energy substrates producing NADH production within 80% of the glucose control. X-axis displays well IDs from the metabolic screening plate (PM-M1).

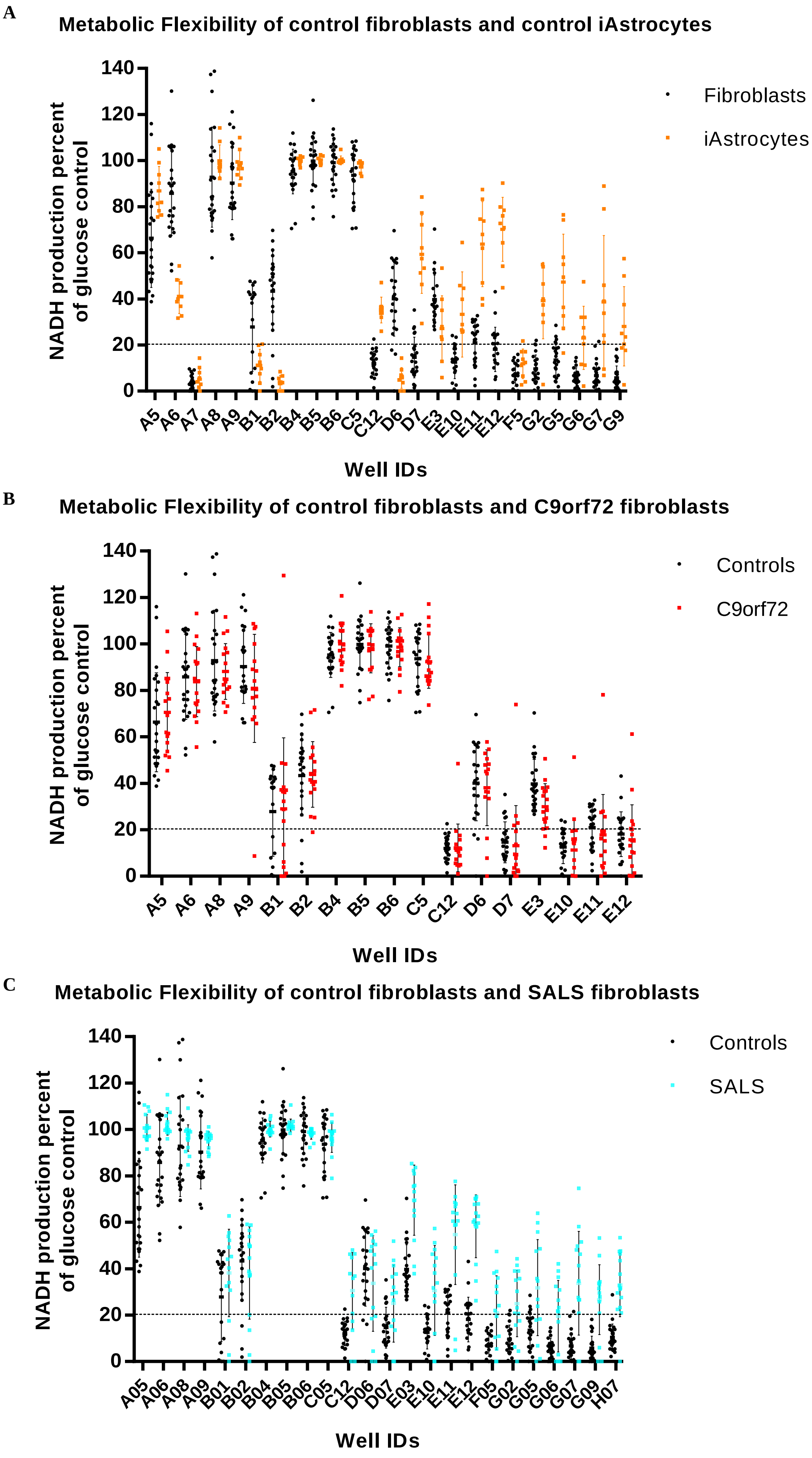
A
Metabolic Flexibility of control fibroblasts and control iAstrocytes
NADH production percent of glucose control
0
20
40
60
80
100
120
140
Fibroblasts
iAstrocytes
A5 A6 A7 A8 A9 B1 B2 B4 B5 B6 C5 C12 D6 D7 E3 E10 E11 E12 F5 G2 G5 G6 G7 G9
Well IDs
B
Metabolic Flexibility of control fibroblasts and C9orf72 fibroblasts
NADH production percent of glucose control
0
20
40
60
80
100
120
140
Controls
C9orf72
A5 A6 A8 A9 B1 B2 B4 B5 B6 C5 C12 D6 D7 E3 E10 E11 E12
Well IDs
C
Metabolic Flexibility of control fibroblasts and SALS fibroblasts
NADH production percent of glucose control
0
20
40
60
80
100
120
140
Controls
SALS
A05 A06 A08 A09 B01 B02 B04 B05 B06 C05 C12 D06 D07 E03 E10 E11 E12 F05 G02 G05 G06 G07 G09 H07
Well IDs

Supplementary Figure 4. Metabolic flexibility is linked to cell survival

(**A**) Cell survival (yellow) vs NADH production (blue) in control iAstrocytes. (**B**) Cell survival (yellow) vs NADH production (blue) in *C9orf72* iAstrocytes. (**C**) Cell survival (yellow) vs NADH production (blue) in SALS iAstrocytes. Data presented as mean with standard deviation of cell survival and NADH production as percent of glucose control. The X-axis displays well IDs from the metabolic screening plate (PM-M1), which corresponds to the position on the 96 well plate, A4 through to H12, each well contains a unique energy substrate apart from B4-B6, which all contain the positive control glucose. A1-A3 are the negative wells and are not included on the graph.

A

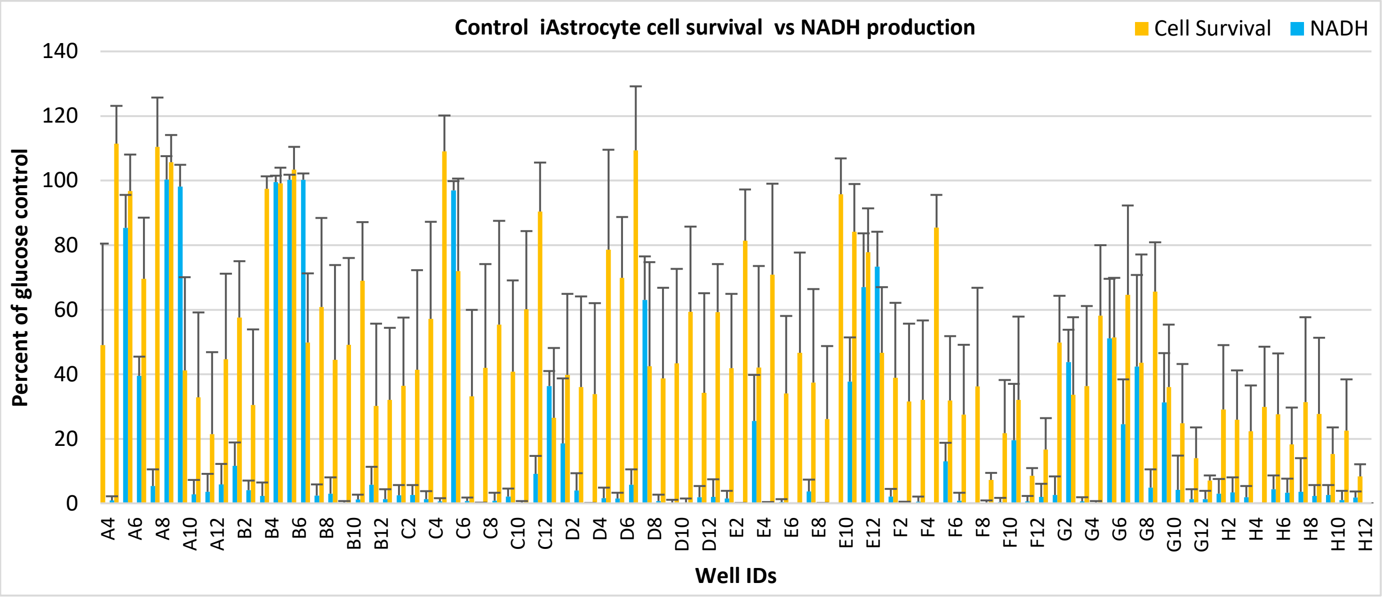
Control iAstrocyte cell survival vs NADH production
Cell Survival
NADH
Percent of glucose control
Well IDs

B

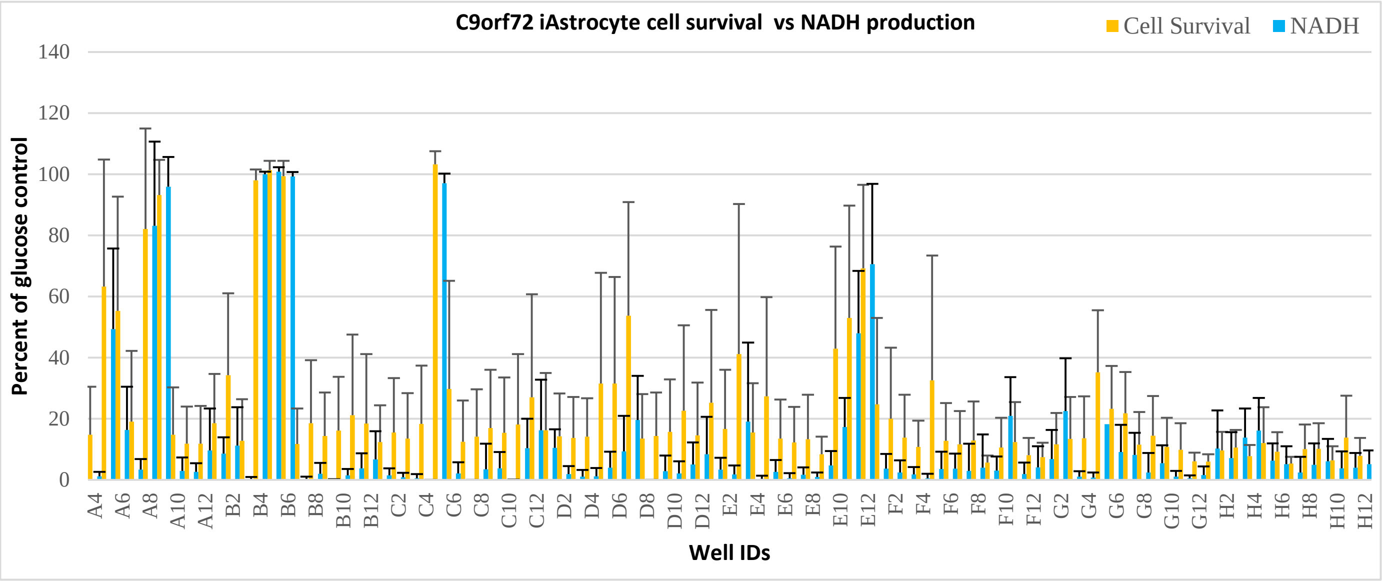
C9orf72 iAstrocyte cell survival vs NADH production
Cell Survival
NADH
Percent of glucose control
Well IDs

C

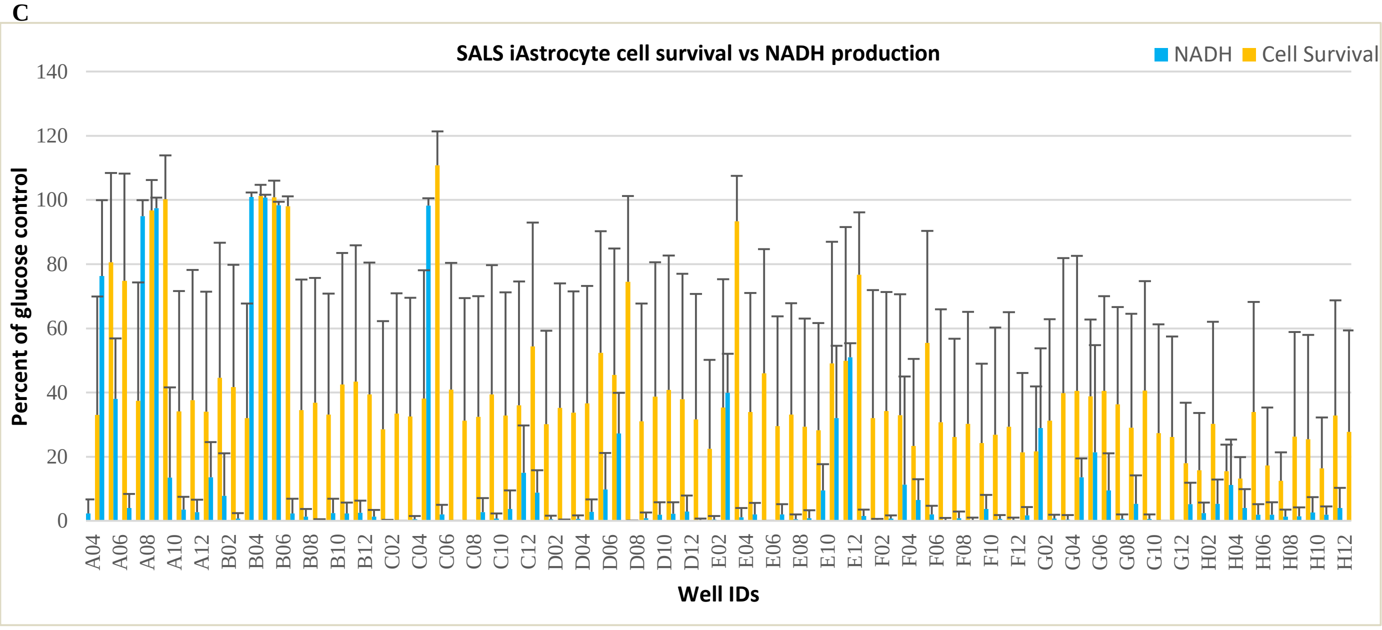
SALS iAstrocyte cell survival vs NADH production
NADH
Cell Survival
Percent of glucose control
Well IDs

Supplementary Figure 5. NADH production kinetic analysis from the iAstrocyte phenotypic metabolic screen plus and minus saponin.

(**A**) NADH production in iAstrocytes with alpha ketoglutaric acid as the sole energy source. (**B**) NADH production in iAstrocytes with alpha ketoglutaric acid as the sole energy source in the presence of saponin. (**C**) NADH production in iAstrocytes with lactic acid as the sole energy source. (**D**) NADH production in iAstrocytes with lactic acid as the sole energy source in the presence of saponin. (**E**) NADH production in iAstrocytes with L-malic acid as the sole energy source. (**F**) NADH production in iAstrocytes with L-malic acid as the sole energy source in the presence of saponin. (**G**) NADH production in iAstrocytes with succinic acid as the sole energy source. (**H**) NADH production in iAstrocytes with succinic acid as the sole energy source in the presence of saponin. (**I**) The effect of saponin on succinic acid induced NADH production in iAstrocytes. Data presented as mean with standard deviation. All data in A-H presented as mean with standard error of 3 controls vs 3 *C9orf72* ALS cases performed in triplicate. Two way ANOVA with AUC and linear regression analysis was performed. * = $P \leq 0.05$. (**J**) iAstrocyte cell number in the presence of cytoplasmic energy substrates. (**K**) iAstrocyte cell number in the presence of mitochondrial energy substrates. Data presented as mean with standard deviation. Data were transformed 1 = 1/Y and 1 = logit (Y) prior to Mann-Whitney analysis. * = $P \leq 0.05$, *** = $P \leq 0.001$.

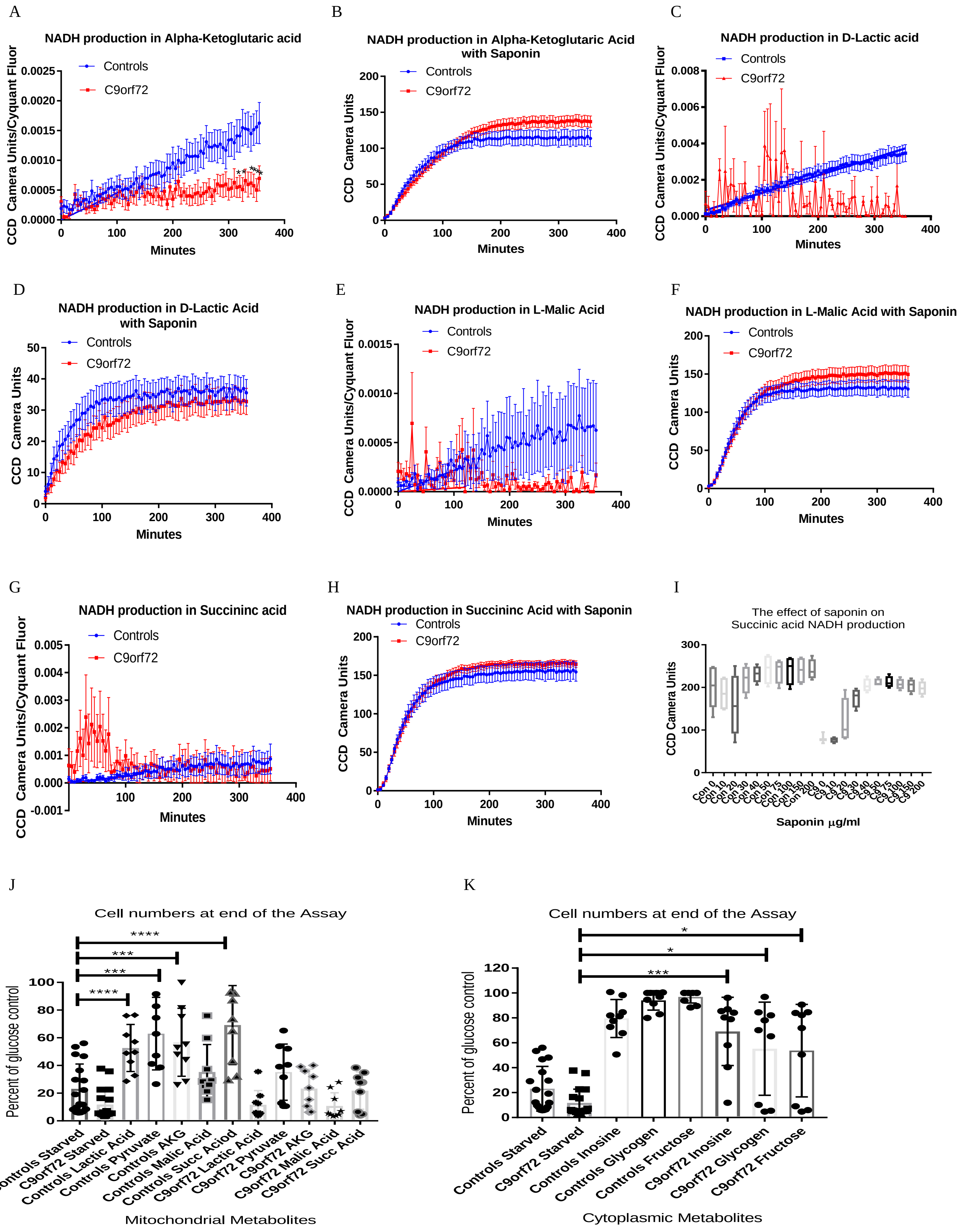

A
NADH production in Alpha-Ketoglutaric acid
CCD Camera Units/Cyquant Fluor
Controls
C9orf72
Minutes
B
NADH production in Alpha-Ketoglutaric Acid with Saponin
CCD Camera Units
Controls
C9orf72
Minutes
C
NADH production in D-Lactic acid
CCD Camera Units/Cyquant Fluor
Controls
C9orf72
Minutes
D
NADH production in D-Lactic Acid with Saponin
CCD Camera Units
Controls
C9orf72
Minutes
E
NADH production in L-Malic Acid
CCD Camera Units/Cyquant Fluor
Controls
C9orf72
Minutes
F
NADH production in L-Malic Acid with Saponin
CCD Camera Units
Controls
C9orf72
Minutes
G
NADH production in Succininc acid
CCD Camera Units/Cyquant Fluor
Controls
C9orf72
Minutes
H
NADH production in Succininc Acid with Saponin
CCD Camera Units
Controls
C9orf72
Minutes
I
The effect of saponin on Succinic acid NADH production
CCD Camera Units
Con 0 Con 10 Con 20 Con 30 Con 40 Con 50 Con 75 Con 100 Con 150 Con 200 C9 0 C9 10 C9 20 C9 30 C9 40 C9 50 C9 75 C9 100 C9 150 C9 200
Saponin µg/ml
J
Cell numbers at end of the Assay
Percent of glucose control
Controls Starved
C9orf72 Starved
Controls Lactic Acid
Controls Pyruvate
Controls AKG
Controls Malic Acid
Controls Succ Aciod
C9orf72 Lactic Acid
C9orf72 Pyruvate
C9orf72 AKG
C9orf72 Malic Acid
C9orf72 Succ Acid
Mitochondrial Metabolites
K
Cell numbers at end of the Assay
Percent of glucose control
Controls Starved
C9orf72 Starved
Controls Inosine
Controls Glycogen
Controls Fructose
C9orf72 Inosine
C9orf72 Glycogen
C9orf72 Fructose
Cytoplasmic Metabolites

Supplementary Figure 6. *C9orf72* iAstrocytes display altered saponin sensitivity compared to controls. Glycogen mobilisation enzyme mRNA levels in *C9orf72* iAstrocytes.

Colour change indicating NADH production in the presence of glucose, L-malic acid and succinic acid and in the presence of increasing saponin concentrations. (**A**) Control 155 vs *C9orf72* case 183. (**B**) Control 3050 vs *C9orf72* case 78. (**C**) Control 209 vs *C9orf72* case 201. A= dye A used in the intact screening assays. C= Dye C used in the permeabilised screening assays. Column 1 no saponin + Dye A. Column 2/3 no saponin + Dye C. Columns 3-12 Dye C with increasing saponin concentration. Data presented as representative images of three independent experiments. (**D**) Glycogen phosphorylase (GP) mRNA levels in *C9orf72* iAstrocytes normalised to U1 snRNA. (**E**) Phosphoglucomutase mRNA levels in *C9orf72* iAstrocytes normalised to U1 snRNA. Data presented as mean and standard deviation from three independent biological replicates. All data analysed by an unpaired t-test analysis. ** = $P \leq 0.01$.

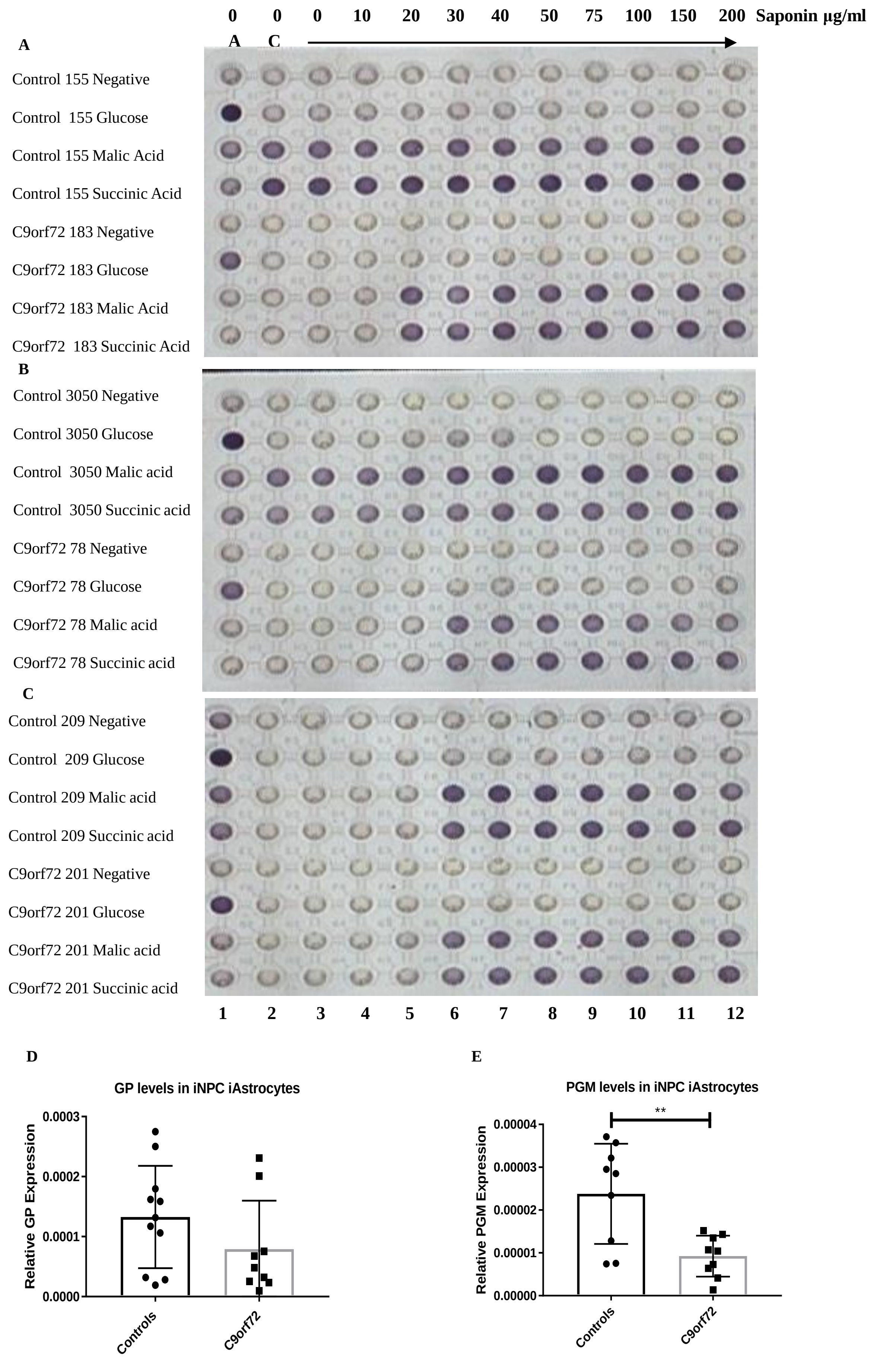
0 0 0 10 20 30 40 50 75 100 150 200 Saponin µg/ml
A C
A
Control 155 Negative
Control 155 Glucose
Control 155 Malic Acid
Control 155 Succinic Acid
C9orf72 183 Negative
C9orf72 183 Glucose
C9orf72 183 Malic Acid
C9orf72 183 Succinic Acid
B
Control 3050 Negative
Control 3050 Glucose
Control 3050 Malic acid
Control 3050 Succinic acid
C9orf72 78 Negative
C9orf72 78 Glucose
C9orf72 78 Malic acid
C9orf72 78 Succinic acid
C
Control 209 Negative
Control 209 Glucose
Control 209 Malic acid
Control 209 Succinic acid
C9orf72 201 Negative
C9orf72 201 Glucose
C9orf72 201 Malic acid
C9orf72 201 Succinic acid
1 2 3 4 5 6 7 8 9 10 11 12
D
GP levels in iNPC iAstrocytes
Relative GP Expression
0.0003
0.0002
0.0001
0.0000
Controls
C9orf72
E
PGM levels in iNPC iAstrocytes
**
Relative PGM Expression
0.00004
0.00003
0.00002
0.00001
0.00000
Controls
C9orf72

Supplementary Figure 7. Glo-1 mRNA levels are reduced in *C9orf72* iAstrocytes.

(**A**) Glo-1 mRNA levels in *C9orf72* iAstrocytes. (**B**) Glo-2 mRNA levels in *C9orf72* iAstrocytes. (**C**) Glo-1 mRNA levels in SALS iAstrocytes. (**D**) Glo-2 mRNA levels in SALS iAstrocytes. (**E**) Densitometry analysis of the protein levels of the targets in sporadic Parkinson's iAstrocyte cases. (**F**) Densitometry analysis of the protein levels of the targets in sporadic Parkinson's fibroblasts cases. (**G**) Representative western blots in sporadic Parkinson's iAstrocyte cases (**H**) Representative western blots in sporadic Parkinson's fibroblast cases (* symbol in PGM western denotes non-specific band). Data presented as mean and standard deviation from three independent biological replicates. A-D, all PCR data normalised to U1 snRNA and analysed by an unpaired t-test analysis. E-F, all western blot data analysed by Kruskal-Wallis with Dunn's post-test analysis. ** = $P \leq 0.01$, *** = $P \leq 0.001$.

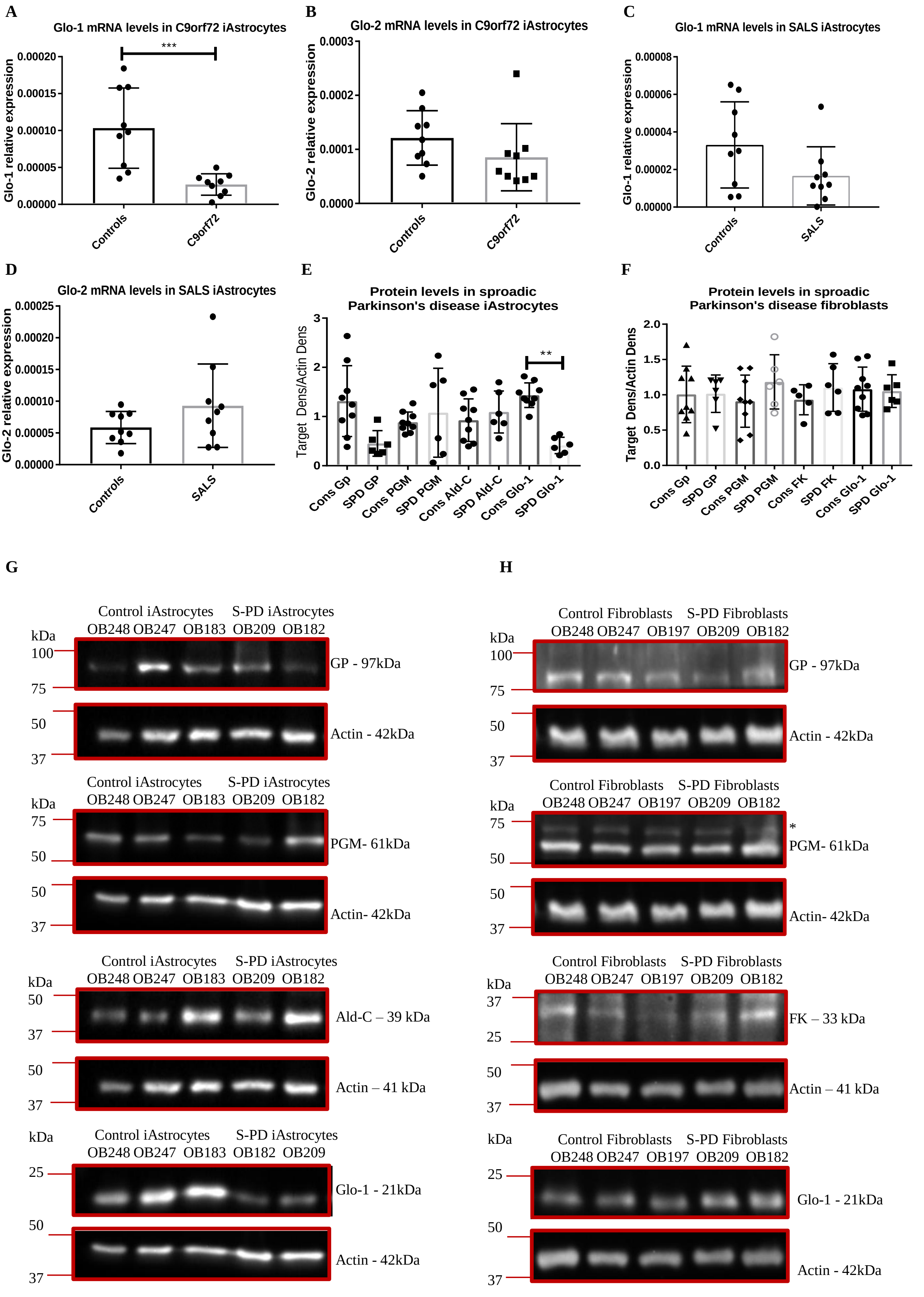
A
Glo-1 mRNA levels in C9orf72 iAstrocytes
Glo-1 relative expression
Controls
C9orf72
B
Glo-2 mRNA levels in C9orf72 iAstrocytes
Glo-2 relative expression
C
Glo-1 mRNA levels in SALS iAstrocytes
SALS
D
Glo-2 mRNA levels in SALS iAstrocytes
E
Protein levels in spradoic Parkinson's disease iAstrocytes
Target Dens/Actin Dens
Cons Gp
SPD GP
Cons PGM
SPD PGM
Cons Ald-C
SPD Ald-C
Cons Glo-1
SPD Glo-1
F
Protein levels in spradoic Parkinson's disease fibroblasts
Cons FK
SPD FK
G
Control iAstrocytes
S-PD iAstrocytes
OB248 OB247 OB183 OB209 OB182
kDa
GP - 97kDa
Actin - 42kDa
PGM- 61kDa
Ald-C – 39 kDa
Actin – 41 kDa
Glo-1 - 21kDa
OB248 OB247 OB183 OB182 OB209
H
Control Fibroblasts
S-PD Fibroblasts
OB248 OB247 OB197 OB209 OB182
FK – 33 kDa

Supplementary Figure 8. Active pyruvate dehydrogenase levels, GLUT1 and GLUT5 in *C9orf72* iAstrocytes are unaltered compared to control.

(A) Representative western blot of pyruvate dehydrogenase (PDH)/ pyruvate dehydrogenase phosphate levels with vinculin as a loading control. (**B**) Densitometry analysis calculating the pyruvate dehydrogenase (active state)/ pyruvate dehydrogenase phosphate (inactive state) ratio. PDH/PDH-P levels were normalised to vinculin before the ratio was calculated by dividing the PDH densitometry values by the PDH-P values. (**C**) iNeuron full length western blots. **(D)** Representative western blot of glucose transporter 1 (GLUT1) with actin as a loading control. (**E**) Densitometry analysis performed by normalising the GLUT1 level to the loading actin. **(F)** Representative western blot of glucose transporter 5 (GLUT5) with actin as a loading control. (**G**) Densitometry analysis performed by normalising the GLUT5 level to the loading actin. For the GLUT5 analysis, cells were treated with 5mM glucose, 0.3mM glutamine -/+ 5mM fructose in DMEM for 24 hours prior to harvesting and western analysis.

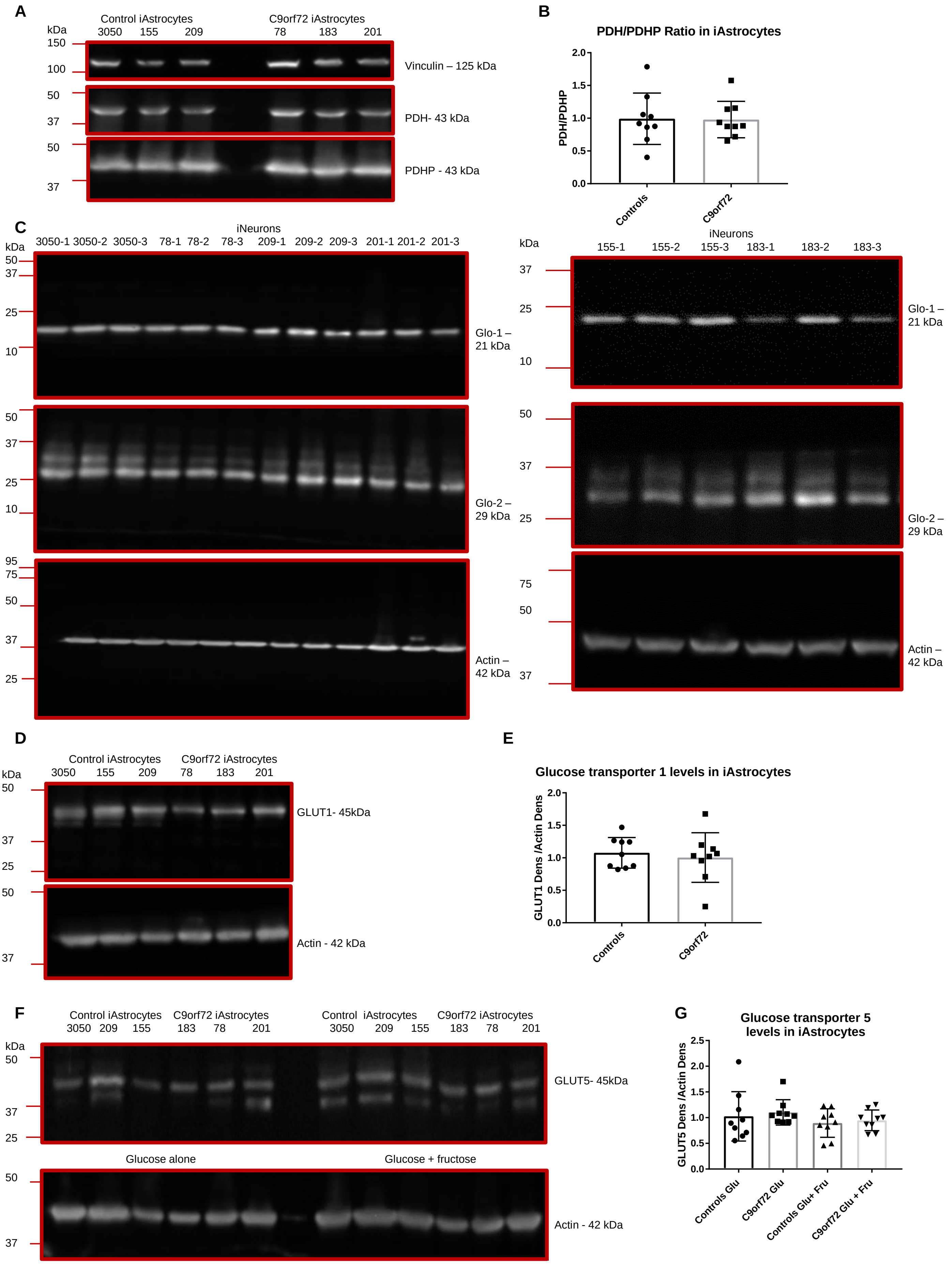
A
Control iAstrocytes
C9orf72 iAstrocytes
kDa
3050 155 209 78 183 201
150
100
50
37
50
37
Vinculin – 125 kDa
PDH- 43 kDa
PDHP - 43 kDa
B
PDH/PDHP Ratio in iAstrocytes
PDH/PDHP
2.0
1.5
1.0
0.5
0.0
Controls
C9orf72
C
iNeurons
kDa
3050-1 3050-2 3050-3 78-1 78-2 78-3 209-1 209-2 209-3 201-1 201-2 201-3
50
37
25
10
Glo-1 – 21 kDa
50
37
25
10
Glo-2 – 29 kDa
95
75
50
37
25
Actin – 42 kDa
iNeurons
kDa
155-1 155-2 155-3 183-1 183-2 183-3
37
25
10
Glo-1 – 21 kDa
50
37
25
Glo-2 – 29 kDa
75
50
37
Actin – 42 kDa
D
Control iAstrocytes
C9orf72 iAstrocytes
kDa
3050 155 209 78 183 201
50
37
25
GLUT1- 45kDa
50
37
Actin - 42 kDa
E
Glucose transporter 1 levels in iAstrocytes
GLUT1 Dens /Actin Dens
2.0
1.5
1.0
0.5
0.0
Controls
C9orf72
F
Control iAstrocytes
C9orf72 iAstrocytes
Control iAstrocytes
C9orf72 iAstrocytes
3050 209 155 183 78 201 3050 209 155 183 78 201
kDa
50
37
25
GLUT5- 45kDa
Glucose alone
Glucose + fructose
50
37
Actin - 42 kDa
G
Glucose transporter 5 levels in iAstrocytes
GLUT5 Dens /Actin Dens
2.5
2.0
1.5
1.0
0.5
0.0
Controls Glu
C9orf72 Glu
Controls Glu+ Fru
C9orf72 Glu + Fru
