# Supplementary material for: C9orf72 expansion within astrocytes reduces metabolic flexibility in amyotrophic lateral sclerosis
Source: Brain. 2019 Oct 24;142(12):3771–90. doi: 10.1093/brain/awz302 (PMC6906594; doi:10.1093/brain/awz302)
Supplement: awz302_Supplementary_Materials [file awz302_supplementary_materials.zip › awz302-suppl_data/Supplementary_Excel_File_1-new.pdf]

Figure	Cell type/Cohort	Conditions	Analysis	Peak Y	Area	Std. Error	95% CI	Slope
Figure 3A	iAstrocyte Controls	Pyruvic Acid	AUC + Linear Regression	0.0000157	0.002322	0.0005857	0.001174 to 0.00347	$5.186 \times 10^{-8} \pm 8.738 \times 10^{-10}$
Figure 3A	iAstrocyte C9orf72	Pyruvic Acid	AUC + Linear Regression	0.00001244	0.001817	0.0005038	0.0008293 to 0.002804	$4.066 \times 10^{-8} \pm 4.02 \times 10^{-10}$
Figure 3B	iAstrocyte Controls	Pyruvic Acid +Saponin	AUC + Linear Regression	29.18	8671	345.4	7994 to 9348	0.09311 ± 0.006021
Figure 3B	iAstrocyte C9orf72	Pyruvic Acid +Saponin	AUC + Linear Regression	27.76	8687	308.9	8082 to 9292	0.09662 ± 0.005334
Figure 3E	iAstrocyte Controls	Negative wells	AUC + Linear Regression	24.17	5578	407.3	4779 to 6376	0.08025 ± 0.002125
Figure 3E	iAstrocyte C9orf72	Negative wells	AUC + Linear Regression	8.333	1653	211.8	1238 to 2068	0.02239 ± 0.0009436
Supplementary Figure 5A	iAstrocyte Controls	Alphaketogutaric acid	AUC + Linear Regression	0.001627	0.2964	0.01958	0.258 to 0.3348	$4.52 \times 10^{-6} \pm 5.179 \times 10^{-8}$
Supplementary Figure 5A	iAstrocyte C9orf72	Alphaketogutaric acid	AUC + Linear Regression	0.0006961	0.1486	0.01409	0.121 to 0.1762	$2.039 \times 10^{-6} \pm 8.257 \times 10^{-8}$
Supplementary Figure 5B	iAstrocyte Controls	Alphaketogutaric acid +Saponin	AUC + Linear Regression	116.4	34299	816.3	32699 to 35899	0.4621 ± 0.01928
Supplementary Figure 5B	iAstrocyte C9orf72	Alphaketogutaric acid +Saponin	AUC + Linear Regression	139.2	37945	603.6	36762 to 39128	0.5331 ± 0.01717
Supplementary Figure 5C	iAstrocyte Controls	Lactic Acid	AUC + Linear Regression	0.00347	0.7215	0.03055	0.6616 to 0.7814	$9.605 \times 10^{-6} \pm 1.859 \times 10^{-7}$
Supplementary Figure 5C	iAstrocyte C9orf72	Lactic Acid	AUC + Linear Regression	0.000687	0.004468	0.003669	0 to 0.01166	$-2.889 \times 10^{-6} \pm 1.127 \times 10^{-6}$
Supplementary Figure 5D	iAstrocyte Controls	Lactic Acid + Saponin	AUC + Linear Regression	37.62	11316	414.1	10504 to 12127	0.1467 ± 0.007047
Supplementary Figure 5D	iAstrocyte C9orf72	Lactic Acid + Saponin	AUC + Linear Regression	34.22	9521	371.9	8792 to 10250	0.129 ± 0.004903
Supplementary Figure 5E	iAstrocyte Controls	L-Malic Acid	AUC + Linear Regression	0.0007726	0.1423	0.02797	0.08751 to 0.1972	$2.163 \times 10^{-6} \pm 4.011 \times 10^{-6}$
Supplementary Figure 5E	iAstrocyte C9orf72	L-Malic Acid	AUC + Linear Regression	0.0006951	0.00876	0.00614	0 to 0.0208	$3.752 \times 10^{-6} \pm 9.118 \times 10^{-8}$
Supplementary Figure 5F	iAstrocyte Controls	L-Malic Acid + Saponin	AUC + Linear Regression	133.1	40437	855.3	38761 to 42114	0.5365 ± 0.0247
Supplementary Figure 5F	iAstrocyte C9orf72	L-Malic Acid + Saponin	AUC + Linear Regression	151.7	44211	836	42572 to 45849	0.6001 ± 0.02433
Supplementary Figure 5G	iAstrocyte Controls	Succininc Acid	AUC + Linear Regression	0.0008737	0.1633	0.02662	0.1111 to 0.2154	$2.479 \times 10^{-6} \pm 4.894 \times 10^{-6}$
Supplementary Figure 5G	iAstrocyte C9orf72	Succininc Acid	AUC + Linear Regression	0.002388	0.2542	0.05599	0.1445 to 0.3639	$2.361 \times 10^{-6} \pm 4.044 \times 10^{-6}$
Supplementary Figure 5H	iAstrocyte Controls	Succininc Acid + Saponin	AUC + Linear Regression	156.6	46685	941.9	44839 to 48531	0.626 ± 0.02715
Supplementary Figure 5H	iAstrocyte C9orf72	Succininc Acid + Saponin	AUC + Linear Regression	166.4	48983	456.5	48088 to 49878	0.6633 ± 0.02739

Supplementary Excel File 1. All Area under the curve and linear regression analysis.
