# Supplementary material for: C9orf72 expansion within astrocytes reduces metabolic flexibility in amyotrophic lateral sclerosis
Source: Brain. 2019 Oct 24;142(12):3771–90. doi: 10.1093/brain/awz302 (PMC6906594; doi:10.1093/brain/awz302)
Supplement: awz302_Supplementary_Materials [file awz302_supplementary_materials.zip › awz302-suppl_data/Supplementary_tables_1_and_2.pdf]

Sample	Type	Sex	Age at biopsy collection (years)	Onset to death (months)
153	Non-ALS control	F	51	-
154	Non-ALS control	F	55	-
155	Non-ALS control	M	40	-
158	Non-ALS control	F	49	-
159	Non-ALS control	F	62	-
209	Non-ALS control	F	69	-
2303	Non-ALS control	M	51	-
3050	Non-ALS control	M	55	-
AG8620	Non-ALS control	F	64	
9	SALS	F	61	21
12	SALS	M	29	90
17	SALS	M	47	72
21	SALS	F	68	16
26	SALS	M	39	32
37	SALS	M	43	49
39	SALS	M	55	-
2191	SALS	F	69	37
46	C9orf72 ALS	F	52	37
53	C9orf72 ALS	F	65	57
78	C9orf72 ALS	M	66	31.7
146	C9orf72 ALS	F	53	31
183	C9orf72 ALS	M	50	27
201	C9orf72 ALS	F	66	19.4
OB183	Non-PD Control	M	50	-
OB197	Non-PD Control	F	50	-
OB247	Non-PD Control	M	58	-
OB248	Non-PD Control	M	55	-
OB182	S-PD	F	51	-
OB209	S-PD	F	56	-

Supplementary Table 1. Details of all control and patient lines used in the study.

	1	2	3	4	5	6	7	8	9	10	11	12
**A**	No Substrate	D-Glucose	Glycogen	D-Glucose-1-PO4	No Substrate	D-Glucose	Glycogen	D-Glucose-1-PO4	No Substrate	D-Glucose	Glycogen	D-Glucose-1-PO4
**B**	D-Glucose-6-PO4	D-Gluconate- 6-PO4	DL-α-Glycerol-PO4	L-Lactic Acid	D-Glucose-6-PO4	D-Gluconate-6-PO4	DL-α-Glycerol-PO4	L-Lactic Acid	D-Glucose-6-PO4	D-Gluconate-6-PO4	DL-α-Glycerol-PO4	L-Lactic Acid
**C**	Pyruvic Acid	Citric Acid	D,L-Isocitric Acid	Cis-Aconitic Acid	Pyruvic Acid	Citric Acid	D,L-Isocitric Acid	Cis-Aconitic Acid	Pyruvic Acid	Citric Acid	D,L-Isocitric Acid	Cis-Aconitic Acid
**D**	α-Keto-Glutaric Acid	Succinic Acid	Fumaric Acid	L-Malic Acid	α-Keto-Glutaric Acid	Succinic Acid	Fumaric Acid	L-Malic Acid	α-Keto-Glutaric Acid	Succinic Acid	Fumaric Acid	L-Malic Acid
**E**	α-Keto-Butyric Acid	D,L,β-Hydroxy-Butyric Acid	L-Glutamic Acid	L-Glutamine	α-Keto-Butyric Acid	D,L,β-Hydroxy-Butyric Acid	L-Glutamic Acid	L-Glutamine	α-Keto-Butyric Acid	D,L,β-Hydroxy-Butyric Acid	L-Glutamic Acid	L-Glutamine
**F**	Ala-Gln	L-Serine	L-Ornithine	Tryptamine	Ala-Gln	L-Serine	L-Ornithine	Tryptamine	Ala-Gln	L-Serine	L-Ornithine	Tryptamine
**G**	L-Malic Acid 100µM	Acetyl-L-Carnitine +L-Malic Acid 100µM	Octanoyl-L-Carnitine +L-Malic Acid 100µM	Palmitoyl-D-L-Carnitine +L-Malic Acid 100µM	L-Malic Acid 100µM	Acetyl-L-Carnitine +L-Malic Acid 100µM	Octanoyl-L-Carnitine +L-Malic Acid 100µM	Palmitoyl-D-L-Carnitine +L-Malic Acid 100µM	L-Malic Acid 100µM	Acetyl-L-Carnitine +L-Malic Acid 100µM	Octanoyl-L-Carnitine +L-Malic Acid 100µM	Palmitoyl-D-L-Carnitine +L-Malic Acid 100µM
**H**	Pyruvic Acid + L-Malic Acid 100µM	Amino-Butyric Acid + L-Malic Acid 100µM	α-Keto-Isocaproic Acid + L-Malic Acid 100µM	L-Leucine+ L-Malic Acid 100µM	Pyruvic Acid + L-Malic Acid 100µM	Amino-Butyric Acid + L-Malic Acid 100µM	α-Keto-Isocaproic Acid + L-Malic Acid 100µM	L-Leucine+ L-Malic Acid 100µM	Pyruvic Acid + L-Malic Acid 100µM	Amino-Butyric Acid + L-Malic Acid 100µM	α-Keto-Isocaproic Acid + L-Malic Acid 100µM	L-Leucine+ L-Malic Acid 100µM

Supplementary Table 2. Layout of the Mitochondrial Function substrate plate, which contains 31 cytosolic and mitochondrial carbon sources in triplicate.
